# Oxysterol binding protein-like 3 (*OSBPL3*) is a novel driver gene that promotes tumor growth in part through R-Ras/Akt signaling in gastric cancer

**DOI:** 10.1038/s41598-021-98485-9

**Published:** 2021-09-28

**Authors:** Qingjiang Hu, Takaaki Masuda, Kensuke Koike, Kuniaki Sato, Taro Tobo, Shotaro Kuramitsu, Akihiro Kitagawa, Atsushi Fujii, Miwa Noda, Yusuke Tsuruda, Hajime Otsu, Yosuke Kuroda, Shuhei Ito, Eiji Oki, Koshi Mimori

**Affiliations:** 1 Department of Surgery, Kyushu University Beppu Hospital, Beppu, 874-0838 Japan; 2 Department of Surgery and Science, Kyushu University Hospital, Fukuoka, 812-8582 Japan; 3 Department of Clinical Laboratory Medicine, Kyushu University Beppu Hospital, Beppu, 874-0838 Japan

**Keywords:** Gastric cancer, Cell growth, Cancer genomics, Gene expression, Cell proliferation

## Abstract

Gastric cancer (GC) is one of the most lethal malignant tumors. To improve the prognosis of GC, the identification of novel driver genes as therapeutic targets is in urgent need. Here, we aimed to identify novel driver genes and clarify their roles in gastric cancer. *OSBPL3* was identified as a candidate driver gene by in silico analysis of public genomic datasets. OSBPL3 expression was analyzed by RT-qPCR and immunohistochemistry in GC cells and tissues. The biological functions and mechanisms of OSBPL3 in GC were examined in vitro and in vivo using GC cells. The association between OSBPL3 expression and clinical outcome in GC patients was also evaluated. Overexpression of OSBPL3 was detected in GC cells with *OSBPL3* DNA copy number gains and promoter hypomethylation. *OSBPL3*-knockdown reduced GC cell growth in vitro and in vivo by inhibiting cell cycle progression. Moreover, an active Ras pull-down assay and western blotting demonstrated that OSBPL3 activates the R-Ras/Akt signaling pathway in GC cells. In a clinical analysis of two GC datasets, high *OSBPL3* expression was predictive of a poor prognosis. Our findings suggest that *OSBPL3* is a novel driver gene stimulating the R-Ras/Akt signaling pathway and a potential therapeutic target in GC patients.

## Introduction

Gastric cancer (GC) is one of the most prevalent cancers worldwide and is associated with a high mortality rate^1,2^. Molecular-targeted therapies have improved the prognosis of various cancers such as colorectal cancer (CRC)^3–5^; however, the number of such therapies for GC is insufficient. Identifying more molecular targets in GC is expected to improve the prognosis of patients.

R-Ras, a member of the Ras oncogene superfamily, plays a key role in multiple cancers including GC^6–10^. Mutations in *RAS* genes can lead to constitutive activation of Ras proteins and subsequent activation of downstream effectors, which are involved in cell growth, differentiation, and survival^11,12^. MAPK/Raf and PI3K/Akt are the main signaling pathways downstream of Ras proteins. It has been reported that R-Ras activates the PI3K/Akt signaling pathway similarly to Kras^13,14^. Interestingly, unlike Kras, few activating mutations in R-Ras have been identified in human cancers, suggesting that R-Ras is activated by other mechanisms such as phosphorylation^7^. Thus, targeting upstream genes that regulate R-Ras activity may be an attractive approach for GC treatment.

Pan-cancer genomic database analyses revealed a positive correlation between the frequency of chromosomal gains and the density of potential driver genes, suggesting that chromosomal amplification is a driving force during cancer development^15^. Recently, we found that amplification of chromosome 7 is a key genomic alteration in CRC revolution^16,17^ and identified eIF5-mimic protein 1 (5MP1)^18^, phosphoserine phosphatase (*PSPH*)^19^, and CRMP5-associated GTPase (*CRAG*)^20^ as novel driver genes located on chromosome 7 in CRC. Notably, integrative analyses of GC with the TCGA dataset showed that chromosome 7 is also ubiquitously and highly amplified in GC^21^. Thus, we hypothesized that chromosome 7 contains driver genes that regulate R-Ras activity in GC.

In this study, we performed in silico analysis and identified oxysterol binding protein-like 3 (*OSBPL3*) as a novel driver gene. *OSBPL3* is located on chromosome 7 that is amplified in GC, and it encodes a protein that phosphorylates the R-Ras protein directly. Next, we examined the biological role and mechanism of OSBPL3 in GC progression using in vitro and in vivo experiments. Finally, we determined the clinical significance of *OSBPL3* expression in not only GC but various solid cancers as well.

## Results

### *OSBPL3* was identified as a candidate driver gene by in silico analysis using the TCGA dataset

**Figure 1:**
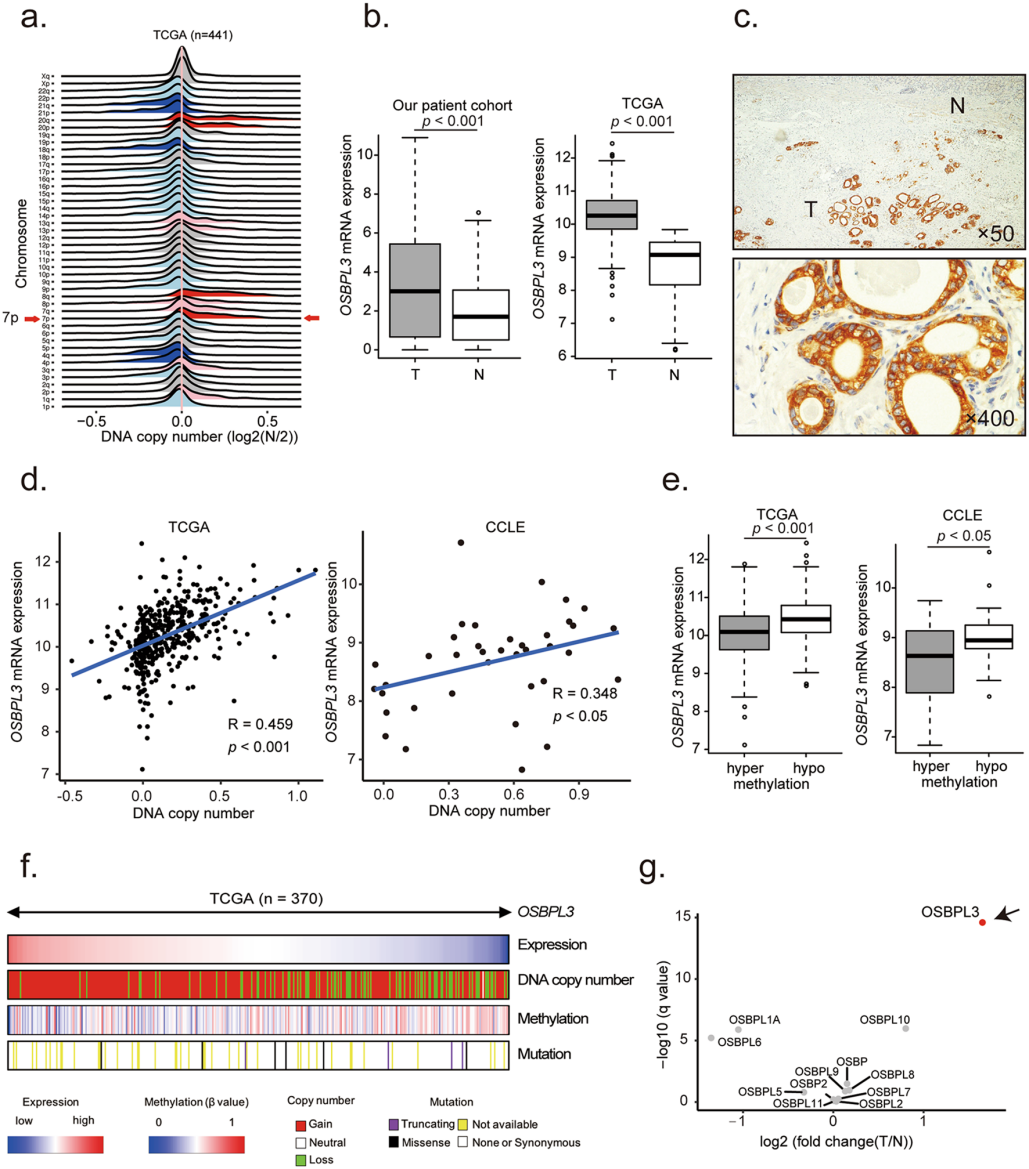
*OSBPL3* expression, DNA copy number, and promoter methylation in GC. (**a**) DNA copy number variations according to chromosome arm in 441 GC tissues from TCGA. (**b**) *OSBPL3* mRNA expression in 109 tumor tissues and 109 paired normal tissues from our GC patient cohort (left panel) and in 415 tumor tissues and 35 normal tissues from GC patients in TCGA (right panel). T: tumor tissue; N: normal tissue. Mann–Whitney U test. (**c**) Immunohistochemical analysis of OSBPL3 in representative samples from our GC patient cohort. Original magnification, × 50 (upper) and ×400 (lower). T: tumor tissue; N: normal tissue. (**d**) Correlation between *OSBPL3* mRNA expression and DNA copy number (log 2(N/2)) in 413 GC patients from TCGA and 38 GC cell lines from the CCLE. (**e**) *OSBPL3* mRNA expression in 185 GC patients exhibiting *OSBPL3* promoter hypomethylation and 185 GC patients exhibiting *OSBPL3* promoter hypermethylation from TCGA (left panel). *OSBPL3* mRNA expression in 17 GC cell lines exhibiting *OSBPL3* promoter hypomethylation and 17 GC cell lines exhibiting *OSBPL3* promoter hypermethylation from the CCLE (right panel). Mann–Whitney U test. (**f**) An integrated view of the mRNA expression, DNA copy number, promoter methylation, and mutation profiles of *OSBPL3* in 370 GC cases from TCGA. The samples are sorted according to the *OSBPL3* mRNA expression level. (**g**) The mRNA expression levels of *OSBP* family members in 415 tumor tissues compared with 35 normal tissues of GC patients from TCGA. Mann–Whitney U test and FDR-controlling procedures were performed.

To identify candidate driver genes in GC based on the TCGA dataset, we used the following three criteria (Supplementary Fig. S1a). First, according to global mRNA expression profiles, the genes of interest are overexpressed in tumor tissues compared with normal tissues of GC patients (Mann–Whitney U test, *q* < 0.05, fold change > 3). Second, using an integrated mRNA expression and DNA copy number profile analysis, the DNA copy number and mRNA expression level of the genes of interest are positively correlated (Pearson’s correlation coefficient > 0.4, *q* < 0.05). Third, the mRNA expression level of the genes of interest are higher than the median mRNA expression level of 20,500 genes from the TCGA dataset. We found 42 genes that satisfied these three criteria. Among these, we selected *OSBPL3* as a candidate driver gene for further analysis because OSBPL3 has the potential to activate the R-Ras signaling pathway^6,7,22^. Notably, the *OSBPL3* gene is located on chromosome 7 that was remarkably amplified in the tumor tissues of GC patients from TCGA (Fig. 1a), suggesting that *OSBPL3* should be a candidate driver gene in GC.

### *OSBPL3* expression was associated with DNA copy number gain and promoter hypomethylation in GC

We first analyzed *OSBPL3* mRNA expression using RT-qPCR in 109 paired tumor and normal tissues from GC patients at our hospital. The expression of *OSBPL3* mRNA was significantly higher in tumor tissues than in normal tissues, consistent with the in silico analysis using the TCGA dataset (Mann–Whitney U test, *p* < 0.001; Fig. 1b). Moreover, immunohistochemical analysis revealed that OSBPL3 staining intensity was stronger in cancer cells than in normal cells (Fig. 1c and Supplementary Fig. S1b). Next, we investigated the mechanism of OSBPL3 upregulation in GC by evaluating the DNA copy number, promoter methylation, and nonsynonymous mutation profiles of *OSBPL3* using TCGA and CCLE datasets. *OSBPL3* expression was positively correlated with the DNA copy number in both datasets (*p* < 0.001 (CCLE) and *p* < 0.05 (TCGA); Fig. 1d). Additionally, 283/370 (76.5%) GC patients from TCGA and 35/38 (92.1%) GC cell lines from CCLE harbored *OSBPL3* DNA copy number gains. Furthermore, high *OSBPL3* expression was also significantly associated with hypomethylation of the *OSBPL3* promoter both in the TCGA and CCLE datasets (Mann–Whitney U test, *p* < 0.001 and *p* < 0.05, respectively; Fig. 1e). To assess the influence of both *OSBPL3* DNA copy number and promoter methylation alterations on *OSBPL3* expression level, we performed multiple linear regression analysis using the TCGA dataset (Fig. 1f). The standardized effect sizes (*t* values) for DNA copy number and promoter methylation alterations were 9.10 (*p* < 0.001) and − 4.03 (*p* < 0.001), respectively. These findings indicated that DNA copy number gain and promoter hypomethylation were independently associated with upregulation of *OSBPL3* expression. There was no significant correlation between *OSBPL3* nonsynonymous mutations and *OSBPL3* expression in GC (Mann–Whitney U test, *p* = 0.168; Supplementary Fig. S1c).

### The expression of OSBP family members in GC

OSBPL3 belongs to the oxysterol binding protein (OSBP) family, which consists of 12 members. Other members of the OSBP family have also been associated with cancer; for example, OSBP2 is overexpressed in T-cell acute lymphoblastic leukemia cells^23^ and essential for cancer cell growth by enhancing Ca^2+^ signaling^24^. We examined the expression of all OSBP family members in GC using the TCGA dataset. Surprisingly, only OSBPL3 was remarkably overexpressed in GC tissues (Fig. 1g), suggesting that OSBPL3 is crucial for GC progression.

### OSBPL3 regulated cell proliferation and tumor growth in GC

**Figure 2:**
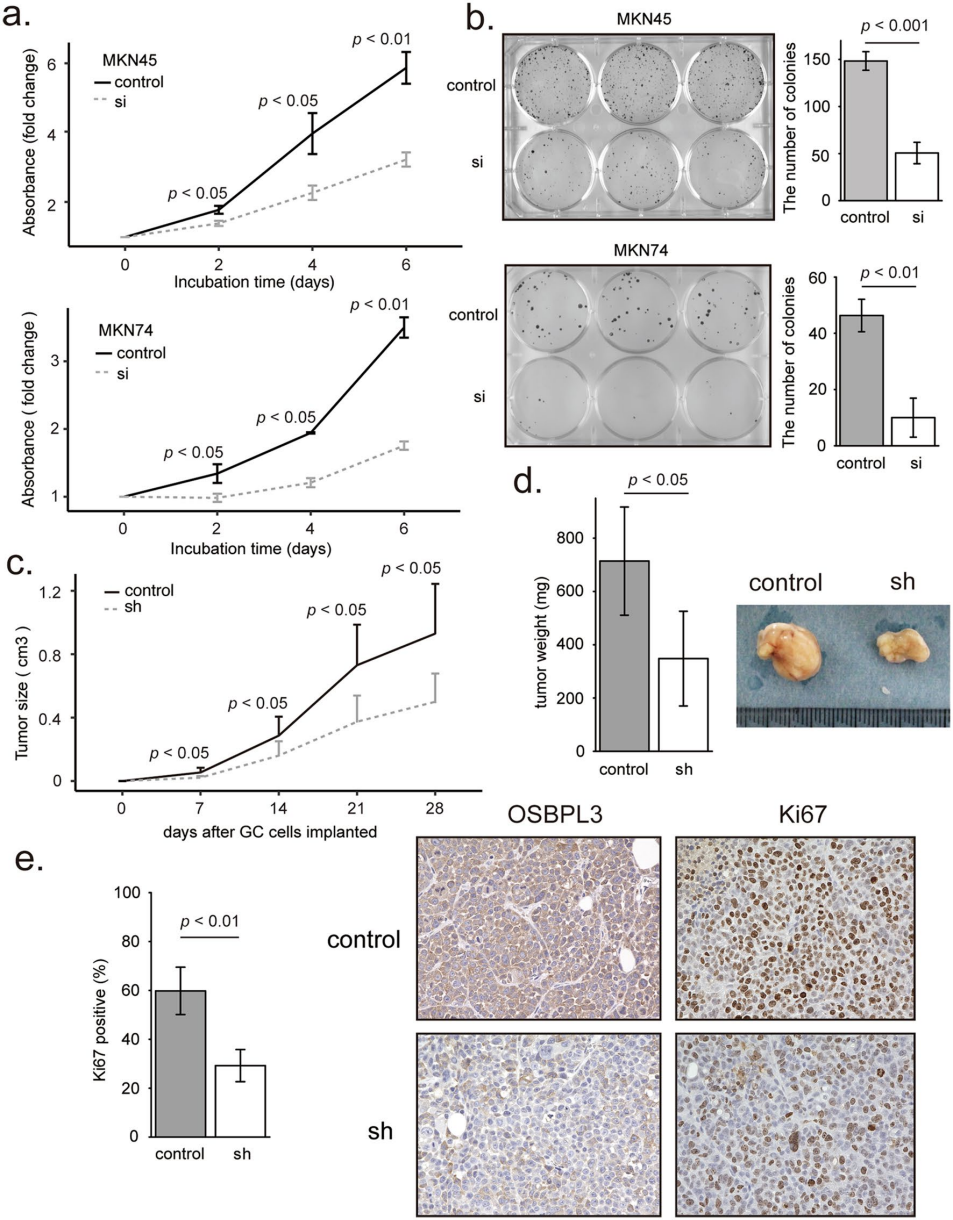
Effect of OSBPL3 on cell proliferation and tumor growth in GC. (**a**) MTT assays in GC cells (MKN45 and MKN74) transfected with *OSBPL3-*specific siRNAs. si: siRNA targeting *OSBPL3*. *N* = 9, error bars represent the mean ± SD. Student’s *t*-test. (**b**) Colony formation assays in GC cells (MKN45 and MKN74) transfected with *OSBPL3-*specific siRNAs. si: siRNA targeting *OSBPL3*. *N* = 3, error bars represent the mean ± SD. Student’s *t*-test. (**c**) Growth curve of tumors derived from 5.0 × 10^6^ GC cells (transfected with *OSBPL3-*specific shRNA or control-shRNA) injected into xenograft mice. sh: shRNA targeting *OSBPL3*. *N* = 7, error bars represent the mean + SD. Student’s *t*-test. (**d**) The weights of tumors derived from 5.0 × 10^6^ GC cells ((transfected with *OSBPL3-*specific shRNA or control-shRNA) injected into xenograft mice and representative images of the tumors. sh: shRNA targeting *OSBPL3*. *N* = 7, error bars represent the mean ± SD. Student’s *t*-test. (**e**) The percentage of Ki67-positive cells in tumors derived from 5.0 × 10^6^ GC cells ((transfected with *OSBPL3-*specific shRNA or control-shRNA) injected into xenograft mice (left panel). *N* = 7, error bars represent the mean ± SD. Immunohistochemical analyses of OSBPL3 and Ki67 in a representative sample from xenograft mice (right panel). sh: shRNA targeting *OSBPL3*. Student’s *t*-test.

OSBPL3 has been reported to activate R-Ras in HEK293 cells^6,7,22^. R-Ras promotes cell proliferation and cell cycle progression through the G1 and S phases^8,25^. R-Ras also activates PI3K/Akt signaling pathway^13,14^, which is a key pathway involved in tumor growth in several cancers^26^. Thus, we hypothesize that OSBPL3 promotes cell proliferation and cell cycle progression by activating the R-Ras/Akt signaling pathway in GC cells. To test this hypothesis, we selected GC cell lines with high *OSBPL3* expression (Supplementary Fig. S2a) and established *OSBPL3*-knockdown MKN45 and MKN74 cells using siRNAs and shRNAs (Supplementary Fig. S2b,c). According to MTT assays, *OSBPL3*-knockdown reduced the proliferation of both MKN45 and MKN74 cells after 6 days (Student’s *t*-test, *p* < 0.01 and *p* < 0.01, respectively; Fig. 2a) (Student’s *t*-test, *p* < 0.01; Supplementary Fig. S3). Colony formation assays showed that the number of colonies was significantly less in *OSBPL3*-knockdown MKN45 and MKN74 cells than in control cells (Student’s *t*-test, *p* < 0.001 and *p* < 0.01, respectively; Fig. 2b). In a xenograft mouse model, tumors derived from *OSBPL3*-knockdown cells were significantly smaller and lower in weight than those derived from control cells (Mann–Whitney U test, n = 7, *p* < 0.05; Fig. 2c,d). Additionally, immunohistochemical analyses revealed that the percentage of Ki67-positive cells was significantly lower in tumors derived from *OSBPL3*-knockdown cells than in those derived from control cells (Mann–Whitney U test, n = 7, *p* < 0.01; Fig. 2e). These results suggest that OSBPL3 is involved in cell proliferation and tumor growth in GC.

### *OSBPL3*-knockdown inhibited cell cycle progression in GC cells

**Figure 3:**
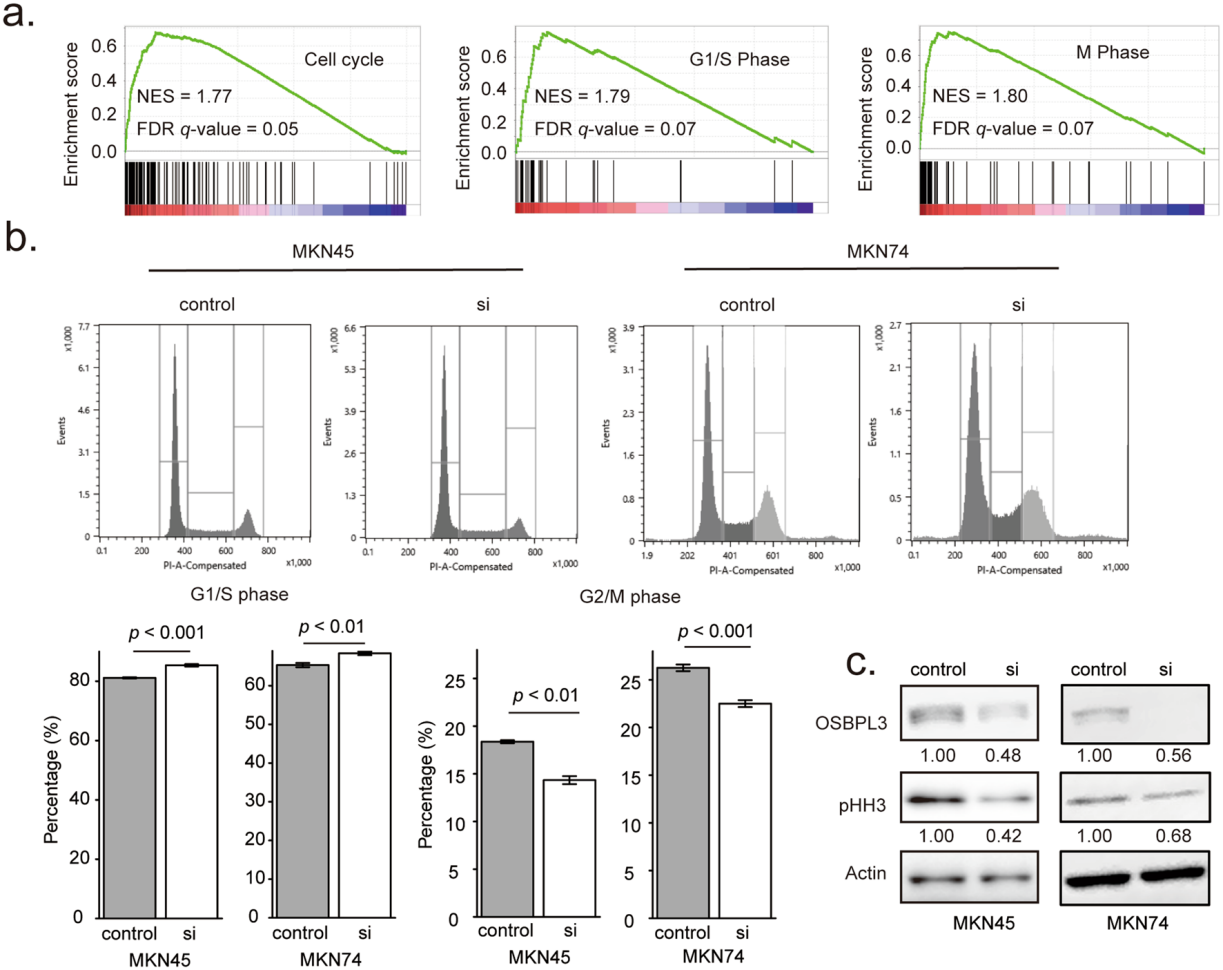
Effect of OSBPL3 on cell cycle progression in GC cells. (**a**) GSEA based on *OSBPL3* mRNA expression in 415 tumor tissues from GC patients in TCGA. (**b**) Representative images of flow cytometry analysis of cell cycle progression in GC cells (upper panels). The bottom panels show the percentages of cells in the G1/S and G2/M phases, respectively, among GC cells (MKN45 and MKN74) transfected with siRNAs. si: siRNA targeting *OSBPL3*. *N* = 3, error bars represent the mean ± SD. Student’s *t*-test. (**c**) The protein levels of OSBPL3, pHH3, and actin in GC cells (MKN45 and MKN74) transfected with siRNAs. si: siRNA targeting *OSBPL3*.

To evaluate the influence of OSBPL3 on cell cycle, we performed GSEA using the TCGA dataset and performed cell cycle analyses using flow cytometry and western blotting. GSEA showed that *OSBPL3* expression was positively correlated with three gene sets associated with cell cycle progression (Fig. 3a). Flow cytometry showed that *OSBPL3*-knockdown significantly increased the proportion of GC cells in the G1 and S phases and decreased the proportion in the G2/M phase (Fig. 3b). Furthermore, western blotting showed a decrease in the level of phosphorylated histone H3 (pHH3) in *OSBPL3*-knockdown GC cells (Fig. 3c), suggesting that the proportion of cells in the M phase was decreased in these cells. These findings indicate that OSBPL3 is involved in maintaining cell cycle progression in GC.

### Knockdown of *OSBPL3* downregulated the R-Ras/Akt signaling pathway

**Figure 4:**
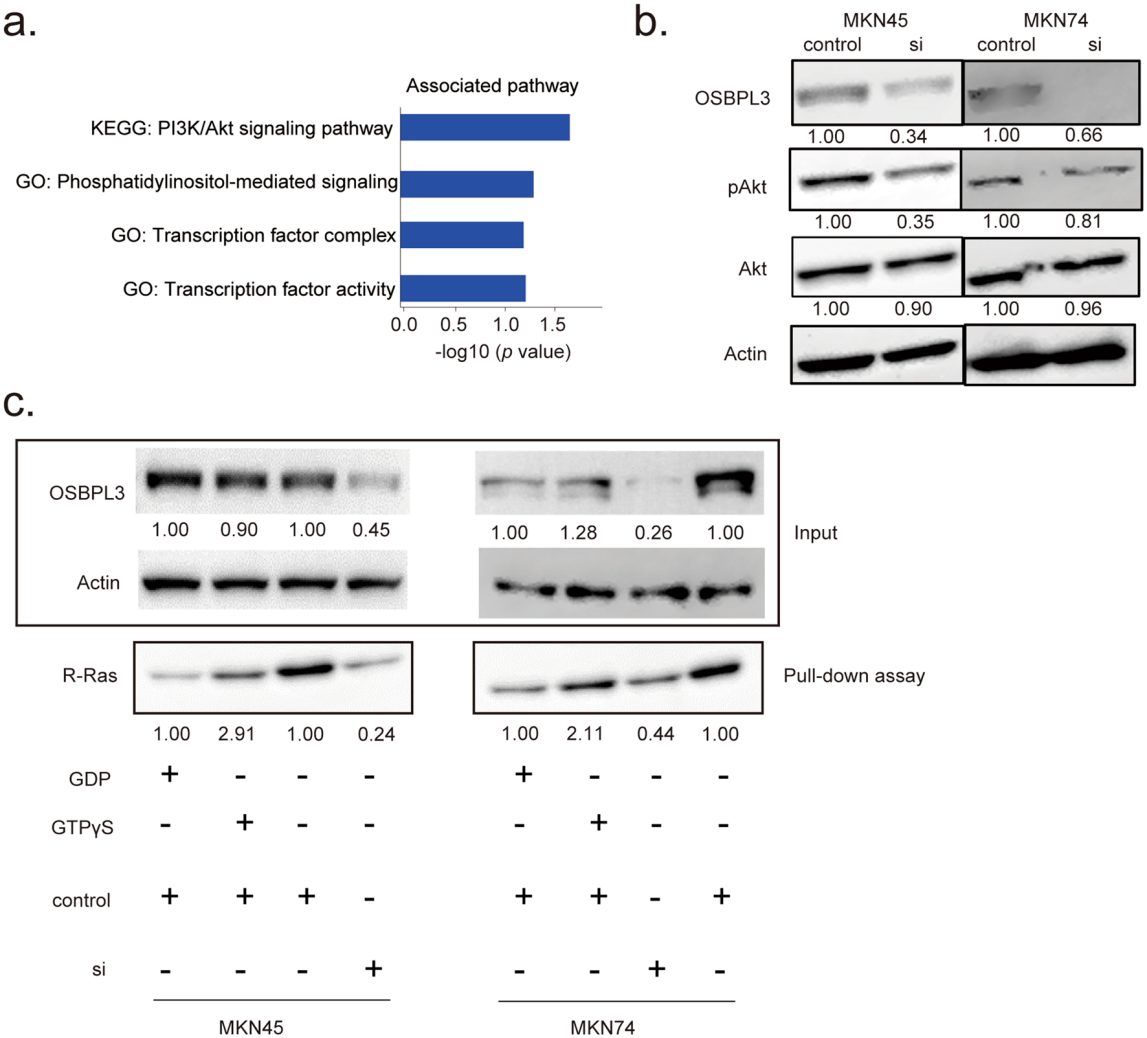
Role of OSBPL3 in activating the R-Ras/Akt signaling pathway in GC cells. (**a**) Pathway analyses of *OSBPL3* gene using the DAVID online tool. (**b**) The protein levels of OSBPL3, pAkt, total Akt, and actin in GC cells (MKN45 and MKN74) transfected with siRNAs. si: siRNA targeting *OSBPL3*. (**c**) Active R-Ras pull-down assay in GC cells (MKN45 and MKN74) transfected with siRNAs. si: siRNA targeting *OSBPL3*.

To investigate whether OSBPL3 can activate the R-Ras/Akt signaling pathway in GC, we first performed pathway analysis based on RNA-seq data from *OSBPL3*-knockdown MKN45 cells. KEGG pathway analysis^27–29^ and Gene Ontology (GO) enrichment showed that OSBPL3 was significantly associated with PI3K/Akt signaling and phosphatidylinositol-mediated signaling in GC cells (Fig. 4a). Next, western blotting showed that the level of pAkt, the activated form of Akt, was reduced in *OSBPL3*-knockdown GC cells (Fig. 4b). Furthermore, an active R-Ras pull-down assay showed that the level of activated R-Ras was reduced in *OSBPL3*-knockdown GC cells (Fig. 4c). These results indicate that the R-Ras/Akt signaling pathway was downregulated in *OSBPL3*-knockdown GC cells.

### The clinical significance of *OSBPL3* expression in GC patients

**Figure 5:**
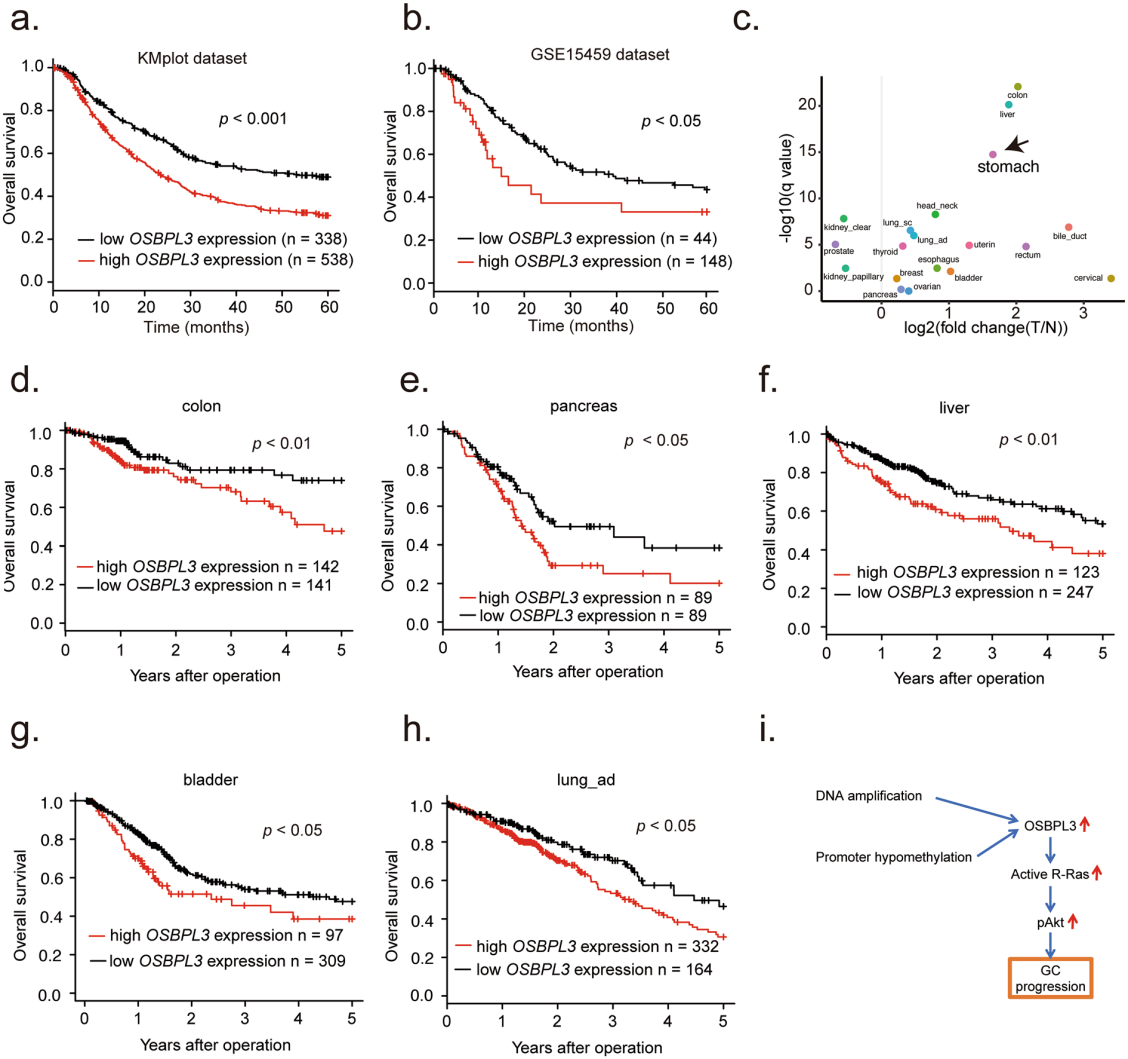
Prognostic significance of *OSBPL3* mRNA expression in GC and other solid cancers. (**a**) Kaplan–Meier survival curve of 876 GC patients from a Kmplot dataset based on *OSBPL3* mRNA expression. Log-rank test. (**b**) Kaplan–Meier survival curve of 192 GC patients from the GSE15459 dataset based on *OSBPL3* mRNA expression. Log-rank test. (**c**) *OSBPL3* mRNA expression in tumor tissues compared with normal tissues in patients with various cancers from TCGA. Mann–Whitney U test and FDR-controlling procedures were performed. (**d**) Kaplan–Meier survival curve of 454 colon cancer patients from TCGA based on *OSBPL3* mRNA expression. Log-rank test. (**e**) Kaplan–Meier survival curve of 178 pancreatic cancer patients from TCGA based on *OSBPL3* mRNA expression. Log-rank test. (**f**) Kaplan–Meier survival curve of 370 liver cancer patients from TCGA based on *OSBPL3* mRNA expression. Log-rank test. (**g**) Kaplan–Meier survival curve of 406 bladder cancer patients from TCGA based on *OSBPL3* mRNA expression. Log-rank test. (**h**) Kaplan–Meier survival curve of 496 lung adenocarcinoma patients from TCGA based on *OSBPL3* mRNA expression. Log-rank test. (**i**) Schematic depicting the mechanisms by which OSBPL3 promotes GC progression.

To evaluate the clinical significance of *OSBPL3* expression in GC, we performed clinicopathological and prognostic analyses using two large GC datasets. The GC cases were divided into high and low *OSBPL3* expression groups, as described in “Materials and methods”. OS was significantly shorter in the high than in the low *OSBPL3* expression group in both the Kmplot dataset (log-rank test, *p* < 0.001; Fig. 5a) and GSE15459 dataset (log-rank test, *p* < 0.05, Fig. 5b). Univariate and multivariate analyses of prognosis were performed next (Table 1). The clinicopathological factors identified as prognostic factors in the univariate analyses were high *OSBPL3* expression, depth of tumor invasion, lymph node metastasis, distant metastasis, and pathological stage. Because depth of tumor invasion, lymph node metastasis, and distant metastasis are highly linked to pathological stage, we selected high *OSBPL3* expression and pathological stage to evaluate in the multivariate analysis. High *OSBPL3* expression was an independent prognostic factor for poor OS in the GSE15459 dataset.

**Table 1:** Univariate and multivariate analyses of clinicopathological factors affecting the overall survival of patients with gastric cancer from the GSE15459 dataset (n = 192).

Variable	Univariate analysis		Multivariate analysis	
	Hazard ratio (CI)	P value	Hazard ratio (CI)	P value
Age (≥ 65 years)	0.89 (0.60–1.34)	0.58		
Female sex	0.71 (0.46–1.09)	0.12		
Histological type (well- or moderately differentiated adenocarcinoma)	0.79 (0.52–1.20)	0.28		
Depth of tumor invasion (≥ SS)	4.75 (2.79–8.63)	< 0.001		
Lymph node metastasis (+)	4.27 (2.31–8.83)	< 0.001		
Distant metastasis (+)	5.23 (3.09–8.62)	< 0.001		
pStage (≥ III)	6.52 (3.73–12.37)	< 0.001	6.55 (3.74–12.43)	< 0.001
Pylori infection (+)	0.74 (0.42–1.31)	0.29		
High *OSBPL3* expression	1.81 (1.09–2.88)	0.02	1.76 (1.06–2.81)	0.03

SS, subserosa; Pylori, helicobacter pylori; pStage, the 6th UICC staging system.

In the clinicopathological analysis, high *OSBPL3* expression was significantly associated with the depth of tumor invasion, lymph node metastasis, stage III/IV, well or moderately differentiated adenocarcinoma, and age (Fisher’s exact test, *p* < 0.05) based on the GSE15459 dataset (Supplementary Table S3). These clinical data indicate that high expression of *OSBPL3* is strongly associated with tumor progression and poor prognosis in GC patients.

### The clinical significance of *OSBPL3* expression across multiple cancers

Finally, we investigated the expression level of *OSBPL3* and its prognostic significance across multiple cancers using TCGA data. *OSBPL3* was overexpressed in tumor tissues compared with normal tissues in almost all cancers evaluated (Fig. 5c), and high *OSBPL3* expression was significantly associated with poor prognosis in colon cancer, pancreatic cancer, liver cancer, bladder cancer, and lung adenocarcinoma (Fig. 5d–h). These findings suggest that *OSBPL3* may be a novel driver gene in all cancer types.

## Discussion

In this study, we identified *OSBPL3* on chromosome 7 as a novel driver gene that facilitates tumor growth by promoting R-Ras/Akt signaling in GC. Furthermore, we found that high expression of *OSBPL3* is an independent biomarker of a poor prognosis in GC. To our knowledge, this is the first study to show that OSBPL3, which is overexpressed by DNA copy number gain and promoter hypomethylation, affects not only the malignant phenotype of GC but also the clinical outcome of GC patients.

OSBPL3 is one of the OSBP family members which are involved in lipid transport^30^, lipid metabolism^31^, and cell signaling^32^. OSBPL3 contains an N-terminal pleckstrin homology (PH) domain and a FFAT (two phenylalanines in an acidic tract) motif. OSBPL3 binds to phosphoinositides via its PH domain^33^ and binds to the ER membrane protein VAPA via its FFAT motif^22^, subsequently stimulating R-Ras signaling, as demonstrated in HEK293 cells^6,7,22^ and colorectal cells^34^. In this study, we found that *OSBPL3*-knockdown reduced the levels of pAkt and active R-Ras in GC cells. These findings are consistent with previous reports and suggest that OSBPL3 can activate the R-Ras/Akt signaling pathway by phosphorylating R-Ras in GC cells.

We found that *OSBPL3*-knockdown decreased tumor growth and inhibited cell cycle progression at the G1 and S phases. GSEA showed that high expression of *OSBPL3* was positively correlated with gene sets associated with cell cycle progression in GC tissues. Furthermore, our clinical analysis showed that high *OSBPL3* expression was correlated with tumor pathological aggressiveness and progression and was an independent factor for a poor prognosis in GC patients. These clinical findings strongly supported the experiments results that OSBPL3 promotes GC cell proliferation. Several lines of evidence have demonstrated that R-Ras promotes cell growth^8,25^ and stimulates cell cycle progression through the G1 phase and subsequent DNA synthesis (S phase)^25^. Therefore, OSBPL3 promotes tumor growth and cell cycle progression at least partially via activation of the R-Ras/Akt signaling pathway in GC (Fig. 5i). Importantly, high expression of OSBPL3 was associated with a DNA copy number gain and promoter hypomethylation, whereas mutations in *OSBPL3* are rare in GC. These findings indicate that OSBPL3 is overexpressed via a gain in DNA copy number and promoter hypomethylation, driving malignancy in GC. Taken together, we provide evidence that *OSBPL3* is a novel driver gene in GC.

Interestingly, some other members of the OSBP family such as OSBP, OSBP2, OSBPL1A, OSBPL6, OSBPL7, and OSBPL9, also contain a PH domain and FFAT motif^35^, which have the potential to bind to phosphoinositides and VAPA and subsequently phosphorylate R-Ras, potential driving malignancy. However, these OSBP genes were not overexpressed in GC tissues. Thus, OSBPL3*,* among all OSBP family members, could play a critical role in GC development. Surprisingly, OSBPL3 was also overexpressed and associated with poor prognosis in colorectal, pancreatic, liver, bladder, and lung cancers in the TCGA database. Hence, OSBPL3 may be essential for driving the development of multiple solid cancers, and targeting OSBPL3 might be a promising therapeutic strategy for not only GC but also various malignancies.

Several driver genes (*5MP1*, *CRAG*, *PSPH*, *GTF2IRD1*, and *DDX56*) were also located on chromosome 7^18–20,36,37^. The expression of these genes should also be increased with *OSBPL3* due to the amplification of chromosome 7 in GC. Thus, these co-amplified driver genes may cooperate with *OSBPL3* in GC progression. Moreover, to our best knowledge, the interaction between *OSBPL3* and other RAS family members is unknown. It is an important issue to examine the interaction in GC progression.

In conclusion, our study demonstrates that *OSBPL3* is a novel driver gene that promotes tumor growth in part by promoting R-Ras/Akt signaling in GC cells. *OSBPL3* may represent a promising therapeutic target for GC. A limitation of this study is that we did not evaluate other mechanisms potentially mediating the effect of OSBPL3 on GC progression. Further investigation is required to clarify this.

## Materials and methods

### Analysis of TCGA

We obtained mRNA expression, DNA copy number alteration, and somatic mutation data from GC and other solid tumors from the Firehose pipeline at the Broad Institute. The detailed data was described in supplementary file 1.

### Cancer cell line encyclopedia (CCLE) dataset

We downloaded mRNA expression data from 1037 cell lines and DNA copy number alteration data from 1042 cell lines in the CCLE. The detailed data was described in supplementary file 1.

### Kmplot dataset

We used KM plotter (http://kmplot.com/gastric), an online tool for survival analyses, to assess the association of *OSBPL3* expression with overall survival (OS) in 876 GC patients^38^. We divided the patients according to the auto-selected best cutoff and selected 5 years as the follow-up time period.

### GSE15459 dataset

The GSE15459 dataset, available from the Gene Expression Omnibus database, consists of mRNA expression and clinical data from 200 GC patients from Singapore. The GC patients were divided according to their *OSBPL3* expression level into high and low expression groups using the minimum *p*-value approach. The detailed data were described in supplementary file 1.

### Gene set enrichment analysis (GSEA)

The associations between *OSBPL3* expression and previously defined gene sets were analyzed by GSEA as described previously^39^, using gene expression profiles of GC patients from TCGA. The biologically defined gene sets were obtained from the Molecular Signatures Database v5.2 (http://software.broadinstitute.org/gsea/msigdb/index.jsp).

### Database for annotation, visualization, and integrated discovery (DAVID)

We used the DAVID online tool (https://david.ncifcrf.gov) for pathway analyses of OSBPL3 in GC cells. The input genes, which were identified as downregulated in MKN45 cells transfected with *OSBPL3-*specific short hairpin RNA (*OSBPL3-*shRNA) relative to control cells (fold change < 0.5), are listed in Supplementary Table S1. The significance of enrichment is expressed as a *p*-value in DAVID.

### Patients and sample collection

This study was approved by the Ethics and Indications Committee of Kyushu University. All research was performed in accordance with relevant guidelines/regulations and with the Helsinki Declaration of 1964 and later versions. Informed consent was obtained from all participants. Paired tumor and normal tissues were obtained from 109 GC patients who provided written informed consent for this study. These patients underwent gastrectomy at Kyushu University Beppu Hospital or an affiliated hospital between 1995 and 2009. The tumor and normal tissues were placed in RNAlater (Takara, Tokyo, Japan), frozen in liquid nitrogen, and stored at − 80 °C. Microdissection of the tumor tissues was not performed because the majority of the tumor tissues comprised cancer cells.

### Antibodies

A rabbit polyclonal antibody against OSBPL3 (Cat# HPA000691) was purchased from Atlas antibodies (Bromma, Sweden). Rabbit polyclonal antibodies against Akt (Cat# 9272), phosphorylated Akt (pAkt, Ser473, Cat# 9271), and R-Ras (Cat# 8446) were all purchased from CST (MA, USA). A mouse monoclonal antibody against actin (Cat# sc-47778) was purchased from Santa Cruz Biotechnology (Santa Cruz, CA, USA). A rabbit polyclonal antibody against Ki67 (Cat# ab15580) and Cell cycle (pCdk/pHH3/Actin) WB cocktail (Cat# ab136810) were purchased from Abcam (Cambridge, UK).

### Immunohistochemical staining

Immunohistochemical analysis of OSBPL3 was performed on formalin-fixed, paraffin-embedded specimens from five GC patients from Kyushu University Hospital using the avidin–biotin–peroxidase method (LSAB2 kit; Dako, Kyoto, Japan). Immunohistochemical analyses of OSBPL3 and Ki67 were performed on tissue specimens from a xenograft mouse model using the same method. All sections were counterstained with hematoxylin. The OSBPL3 and Ki67 primary antibodies were used at dilutions of 1:100 and 1:1000, respectively. Histological analysis was independently performed by an experienced research pathologist at Kyushu University.

### Western blotting

Total proteins were extracted from the samples using lysis buffer. Western blotting was performed as described previously^40,41^ using the following specific primary antibodies (dilution): OSBPL3 (1:350), Akt (1:1000), pAkt (Ser473) (1:1000), R-Ras (1:1000), actin (1:1000), and Cell cycle (pCdk/pHH3/Actin) WB cocktail (1:250). The blots were then incubated with horseradish peroxidase-conjugated anti-rabbit or anti-mouse immunoglobulin (Promega, WI, USA). Signals were detected using Immobilon (Millipore, MA, US).

### Cell lines and cell culture

Human GC cell lines (MKN45 (Cat# JCRB0254), MKN74 (Cat# JCRB0255) were purchased from the Japanese Collection of Research Bioresources Cell Bank (JCRB), National Institutes of Biomedical Innovation, Health and Nutrition, Japan. These cell lines have been tested and authenticated using STR-PCR method by JCRB. These cell lines were passaged immediately after receipt in our laboratory for this study. These cell lines were cultured in RPMI 1640 medium (Gibco, CA, USA) supplemented with 10% fetal bovine serum at 37 °C in a humidified atmosphere containing 5% CO_2_.

### Extraction of total RNA

Total RNA was extracted from subconfluent cell cultures using ISOGEN (NIPPON GENE, Tokyo, Japan) according to the manufacturer’s instructions.

### Reverse transcription quantitative PCR (RT-qPCR)

Reverse transcription was performed using M-MLV Reverse Transcriptase (Invitrogen) as described previously^39^. qPCR was performed using LightCycler 480 SYBR Green I Master Mix (Roche, Basel, Swiss) according to the manufacturer’s instructions. The following primers were used: *OSBPL3*, 5′-GTGGCCCTTAAAAGGCTGGC-3′ (sense) and 5′-GAGCCCGACATCAATGCAGC-3′ (antisense); *GAPDH*, 5′-AGCCACATCGCTCAGACAC-3′ (sense) and 5′-GCCCAATACGACCAAATCC-3′ (antisense); The mRNA level of *OSBPL3* was normalized to that of *GAPDH*.

### RNA sequencing (RNA-seq)

RNA-seq was performed using the Illumina HiSeq 2500 platform, conducted at BGI (Beijing, China). We sent total RNA extracted from *OSBPL3*- or control-shRNA-transfected MKN45 cells (n = 2) to BGI Japan and received fastq files of RNA-seq reads. The reads were aligned to the human reference genome, and genes were annotated (UCSC hg19) using TopHat2 v2.0.12. Cufflinks v2.2.1 was used to calculate the FPKM values of each gene.

### Transfection assays and establishment of GC cells stably transfected with *OSBPL3*-shRNA

To achieve transient knockdown of *OSBPL3*, GC cells were transfected with siRNAs targeting *OSBPL3* using Lipofectamine RNAiMAX (Invitrogen), according to the manufacturer’s instructions. Two individual *OSBPL3* siRNAs (siOSBPL3 #1 and siOSBPL3 #2) and Silencer Negative Control No. 1 siRNA were purchased from Invitrogen. The backbone plasmid pcDNA6.2-GW/EmGFP-miR was obtained from the Block-iT Pol II miR RNAi Expression Vector Kit (Invitrogen). The plasmids pcDNA6.2-GW/EmGFP-*OSBPL3*-shRNA, containing three individual *OSBPL3*-shRNAs (shOSBPL3 #1, shOSBPL3 #2, and shOSBPL3 #3), and pcDNA6.2-GW/EmGFP-control-shRNA (control-shRNA), containing an unrelated insert, were constructed according to the manual of the Block-iT Pol II miR RNAi Expression Vector Kit. *OSBPL3*-shRNAs and control-shRNA were transfected into MKN45 cells using Lipofectamine 3000 (Invitrogen) according to the manufacturer’s instructions. Stably transfected cells were selected using blasticidin (6 μg/mL), followed by sorting for GFP by FACS. A clone transfected with control-shRNA was used as the control. The sequences of the siRNAs and shRNAs targeting *OSBPL3* are listed in Supplementary Table S2.

### MTT assay

Cell proliferation was evaluated by MTT assay using the Cell Proliferation Kit 1 (Roche Applied Science, Penzberg, Germany) according to the manufacturer’s instructions. In brief, transfected cells (MKN45 and MKN74) were seeded at 10,000 cells/well in triplicate wells of a 24-well plate in 500 μL medium. The color reaction was quantitated using an automatic plate reader, Immuno-Mini NJ-2300 (Nihon InterMed, Tokyo, Japan), at 570 nm with a reference filter of 650 nm. Each independent experiment was performed three times.

### Colony formation assay

Transfected cells were seeded at 1000 cells/well in triplicate wells of a six-well plate and maintained in the appropriate medium containing 10% fetal bovine serum for 2 weeks. At 14 days, the cells were fixed and stained with Diff-Quick (Sysmex, Kobe, Japan). Visible colonies were then counted using Fusion SOLO S software (Vilber Lourmat, Paris, France). Each independent experiment was performed three times.

### Cell cycle assay

Propidium iodide staining was performed to assess the cell cycle. MKN45 and MKN74 cells were transfected with siOSBPL3 and harvested by trypsinization at 48 h after transfection. The cells were fixed in 3 mL cold 70% ethanol and then stained with propidium iodide (Wako, Osaka, Japan) for 30 min at room temperature. We then analyzed the cells by flow cytometry (Sony Biotechnology, Tokyo, Japan). According to their DNA content, the cells were assigned to the G1, S, and G2M phases, and then the relative proportion of cells in each phase was compared with that of the control transfectants in each experiment.

### Active R-Ras pull-down assay

MKN45 and MKN74 cells transfected with *OSBPL3* siRNA or Silencer Negative Control No. 1 siRNA were lysed and subjected to GST-Raf-RBD pull-down assays using the Active Ras Detection Kit (CST) according to the manufacturer’s instructions. Lysates (input) and pull-down material were analyzed by western blotting. The pull-down assays were performed in triplicate according to the manufacturer’s protocol. The main procedure is as follows. (1) Lysates of MKN45 and MKN74 cells were prepared with 1× lysis buffer. (2) Cell lysates (control cells with GTPγS or GDP treatments: 500 μg/sample, control cells without treatment and siRNA cells: 700 μg/sample), binding protein, and glutathione resin in the spin cup and incubate at 4 °C to allow GTP-bound GTPase binding to the glutathione resin through GST-linked binding protein. (3) Remove unbound proteins by centrifugation. (4) Elute glutathione resin-bound GTPase with SDS buffer. (5) The eluted samples were analyzed by western blot using an R-Ras antibody.

### Xenograft mouse model

To analyze the effects of OSBPL3 on tumorigenesis and tumor growth, we established a xenograft mouse model using 6- to 8-week-old BALB/c nu/nu female mice. MKN45 cells were stably transfected with *OSBPL3*-shRNA or control-shRNA in 10 cm culture plates, harvested and washed with PBS, and suspended at a concentration of 5.0 × 10^6^ cells/mL. A total of 200 μL suspended cells was injected subcutaneously into a single side of the posterior flank of each mouse. *OSBPL3*-shRNA- and control-shRNA-transfected cells were injected into the right and left flank sides of the same mouse, respectively. Tumor growth was examined every week, and tumor volumes were calculated using the following equation: V = D × d × H (V, volume; D, longest diameter; d, diameter perpendicular to the longest diameter; H, height). At 28 days postinjection, the mice were euthanized. We measured the weight of each subcutaneous tumor and examined the tumor tissues by immunohistochemical analyses. All of the animal studies were approved by the ethics committee of Kyushu University, and all animal procedures were performed in compliance with the Guidelines for the Care and Use of Experimental Animals established by the Committee for Animal Experimentation of Kyushu University; these guidelines conform to the ethical standards required by Japanese law and also comply with the guidelines for the use of experimental animals in Japan. The study involving animals was also carried out in compliance with the ARRIVE guidelines.

### Statistical analysis

Associations between variables were assessed using the Mann–Whitney U test, Student’s *t*-test, or Chi-squared test, where appropriate. The degree of linearity was estimated by Pearson’s correlation coefficient. OS was estimated using the Kaplan–Meier method, and survival curves were compared using the log-rank test. Univariate and multivariate analyses were performed using the Cox regression model to identify independent variables predictive of OS. A two-sided P < 0.05 was considered significant. For multiple comparisons, FDR-controlling procedures were performed using the Benjamini–Hochberg method, and a *q*-value < 0.05 was considered significant. Data analyses were performed using JMP Pro 13 software (SAS Institute, Cary, NC, USA) and R software version 3.3.2 (The R Foundation).

## Supplementary Information

Supplementary Information.

## References

[1] Sung H, Ferlay J, Siegel RL, Laversanne M, Soerjomataram I, Jemal A (2021). Global cancer statistics 2020: GLOBOCAN estimates of Incidence and mortality worldwide for 36 cancers in 185 countries. CA Cancer J. Clin..

[2] Torre LA, Bray F, Siegel RL, Ferlay J, Lortet-Tieulent J, Jemal A (2015). Global cancer statistics, 2012. CA Cancer J. Clin..

[3] Van Cutsem E, Kohne CH, Hitre E, Zaluski J, Chien CRC, Makhson A (2009). Cetuximab and chemotherapy as initial treatment for metastatic colorectal cancer. N. Engl. J. Med..

[4] Swain SM, Kim SB, Cortes J, Ro J, Semiglazov V, Campone M (2013). Pertuzumab, trastuzumab, and docetaxel for HER2-positive metastatic breast cancer (CLEOPATRA study): Overall survival results from a randomised, double-blind, placebo-controlled, phase 3 study. Lancet Oncol..

[5] Pirker R, Pereira JR, Szczesna A, von Pawel J, Krzakowski M, Ramlau R (2009). Cetuximab plus chemotherapy in patients with advanced non-small-cell lung cancer (FLEX): An open-label randomised phase III trial. Lancet.

[6] Nishigaki M, Aoyagi K, Danjoh I, Fukaya M, Yanagihara K, Sakamoto H (2005). Discovery of aberrant expression of R-RAS by cancer-linked DNA hypomethylation in gastric cancer using microarrays. Cancer Res..

[7] Fremin C, Guegan JP, Plutoni C, Mahaffey J, Philips MR, Emery G (2016). ERK1/2-induced phosphorylation of R-Ras GTPases stimulates their oncogenic potential. Oncogene.

[8] Yu Y, Feig LA (2002). Involvement of R-Ras and Ral GTPases in estrogen-independent proliferation of breast cancer cells. Oncogene.

[9] Mora N, Rosales R, Rosales C (2007). R-Ras promotes metastasis of cervical cancer epithelial cells. Cancer Immunol. Immunother..

[10] Chen Y, Soong J, Mohanty S, Xu L, Scott G (2013). The neural guidance receptor Plexin C1 delays melanoma progression. Oncogene.

[11] Downward J (2003). Targeting RAS signalling pathways in cancer therapy. Nat. Rev. Cancer..

[12] Schubbert S, Shannon K, Bollag G (2007). Hyperactive Ras in developmental disorders and cancer. Nat. Rev. Cancer..

[13] Marte BM, Rodriguez-Viciana P, Wennström S, Warne PH, Downward J (1997). R-Ras can activate the phosphoinositide 3-kinase but not the MAP kinase arm of the Ras effector pathways. Curr. Biol..

[14] Osada M, Tolkacheva T, Li W, Chan TO, Tsichlis PN, Saez R (1999). Differential roles of Akt, Rac, and Ral in R-Ras-mediated cellular transformation, adhesion, and survival. Mol. Cell Biol..

[15] Davoli T, Xu AW, Mengwasser KE, Sack LM, Yoon JC, Park PJ (2013). Cumulative haploinsufficiency and triplosensitivity drive aneuploidy patterns and shape the cancer genome. Cell.

[16] Uchi R, Takahashi Y, Niida A, Shimamura T, Hirata H, Sugimachi K (2016). Integrated multiregional analysis proposing a new model of colorectal cancer evolution. PLoS Genet..

[17] Saito T, Niida A, Uchi R, Hirata H, Komatsu H, Sakimura S (2018). A temporal shift of the evolutionary principle shaping intratumor heterogeneity in colorectal cancer. Nat Commun..

[18] Sato K, Masuda T, Hu Q, Tobo T, Gillaspie S, Niida A (2019). Novel oncogene 5MP1 reprograms c-Myc translation initiation to drive malignant phenotypes in colorectal cancer. EBioMedicine.

[19] Sato K, Masuda T, Hu Q, Tobo T, Kidogami S, Ogawa Y (2017). Phosphoserine phosphatase is a novel prognostic biomarker on chromosome 7 in colorectal cancer. Anticancer Res..

[20] Shimizu D, Masuda T, Sato K, Tsuruda Y, Otsu H, Kuroda Y (2019). CRMP5-associated GTPase (CRAG) Is a candidate driver gene for colorectal cancer carcinogenesis. Anticancer Res..

[21] Cancer Genome Atlas Research N (2014). Comprehensive molecular characterization of gastric adenocarcinoma. Nature.

[22] Weber-Boyvat M, Kentala H, Lilja J, Vihervaara T, Hanninen R, Zhou Y (2015). OSBP-related protein 3 (ORP3) coupling with VAMP-associated protein A regulates R-Ras activity. Exp Cell Res..

[23] Zhong W, Yi Q, Xu B, Li S, Wang T, Liu F (2016). ORP4L is essential for T-cell acute lymphoblastic leukemia cell survival. Nat Commun..

[24] Li JW, Xiao YL, Lai CF, Lou N, Ma HL, Zhu BY (2016). Oxysterol-binding protein-related protein 4L promotes cell proliferation by sustaining intracellular Ca2+ homeostasis in cervical carcinoma cell lines. Oncotarget.

[25] Self AJ, Caron E, Paterson HF, Hall A (2001). Analysis of R-Ras signalling pathways. J Cell Sci..

[26] Vivanco I, Sawyers CL (2002). The phosphatidylinositol 3-Kinase AKT pathway in human cancer. Nat Rev Cancer..

[27] Kanehisa M, Goto S (2000). KEGG: Kyoto encyclopedia of genes and genomes. Nucleic Acids Res..

[28] Kanehisa M (2019). Toward understanding the origin and evolution of cellular organisms. Protein Sci..

[29] Kanehisa M, Furumichi M, Sato Y, Ishiguro-Watanabe M, Tanabe M (2021). KEGG: Integrating viruses and cellular organisms. Nucleic Acids Res..

[30] Du X, Turner N, Yang H (2017). The role of oxysterol-binding protein and its related proteins in cancer. Semin Cell Dev Biol..

[31] Yan D, Lehto M, Rasilainen L, Metso J, Ehnholm C, Yla-Herttuala S (2007). Oxysterol binding protein induces upregulation of SREBP-1c and enhances hepatic lipogenesis. Arterioscler Thromb Vasc Biol..

[32] Lehto M, Mayranpaa MI, Pellinen T, Ihalmo P, Lehtonen S, Kovanen PT (2008). The R-Ras interaction partner ORP3 regulates cell adhesion. J Cell Sci..

[33] Lehto M, Hynynen R, Karjalainen K, Kuismanen E, Hyvarinen K, Olkkonen VM (2005). Targeting of OSBP-related protein 3 (ORP3) to endoplasmic reticulum and plasma membrane is controlled by multiple determinants. Exp Cell Res..

[34] Jiao HL, Weng BS, Yan SS, Lin ZM, Wang SY, Chen XP (2020). Upregulation of OSBPL3 by HIF1A promotes colorectal cancer progression through activation of RAS signaling pathway. Cell Death Dis..

[35] Zhou Y, Wohlfahrt G, Paavola J, Olkkonen VM (2014). A vertebrate model for the study of lipid binding/transfer protein function: Conservation of OSBP-related proteins between zebrafish and human. Biochem Biophys Res Commun..

[36] Nambara S, Masuda T, Kobayashi Y, Sato K, Tobo T, Koike K (2020). GTF2IRD1 on chromosome 7 is a novel oncogene regulating the tumor-suppressor gene TGFβR2 in colorectal cancer. Cancer Sci..

[37] Kouyama Y, Masuda T, Fujii A, Ogawa Y, Sato K, Tobo T (2019). Oncogenic splicing abnormalities induced by DEAD-Box Helicase 56 amplification in colorectal cancer. Cancer Sci..

[38] Szasz AM, Lanczky A, Nagy A, Forster S, Hark K, Green JE (2016). Cross-validation of survival associated biomarkers in gastric cancer using transcriptomic data of 1,065 patients. Oncotarget.

[39] Uchi R, Kogo R, Kawahara K, Sudo T, Yokobori T, Eguchi H (2013). PICT1 regulates TP53 via RPL11 and is involved in gastric cancer progression. Br J Cancer..

[40] Kurashige J, Hasegawa T, Niida A, Sugimachi K, Deng N, Mima K (2016). Integrated Molecular Profiling of Human Gastric Cancer Identifies DDR2 as a Potential Regulator of Peritoneal Dissemination. Sci Rep..

[41] Ueo H, Sugimachi K, Gorges TM, Bartkowiak K, Yokobori T, Muller V (2015). Circulating tumour cell-derived plastin3 is a novel marker for predicting long-term prognosis in patients with breast cancer. Br J Cancer..

