# Supplementary material for: Oxysterol binding protein-like 3 (OSBPL3) is a novel driver gene that promotes tumor growth in part through R-Ras/Akt signaling in gastric cancer
Source: Sci Rep. 2021 Sep 28;11:19178. doi: 10.1038/s41598-021-98485-9 (PMC8478956; doi:10.1038/s41598-021-98485-9)

SUPPLEMENTARY FIGURE LEGENDS

Supplementary Figure S1. Identification of *OSBPL3* as a potential driver gene in GC.

a. Schematic summarizing our identification of *OSBPL3* as a candidate driver gene using TCGA data.

b. Immunohistochemical analysis of OSBPL3 in five representative samples from our GC patient cohort. Original magnification, ×50 (upper) and ×400 (lower). T: tumor tissue; N: normal tissue.

c. *OSBPL3* mRNA expression in 10 GC patients harboring *OSBPL3* nonsynonymous mutations and in 360 GC patients from TCGA.

Supplementary Figure S2. Knockdown of *OSBPL3* using siRNAs and shRNAs.

a. *OSBPL3* mRNA and protein expression in nine gastric cancer (GC) cell lines according to RT-qPCR (upper panel) and western blotting (bottom panel), respectively. $N = 3$, error bars represent the mean ± SD.

b. *OSBPL3* mRNA and protein expression in GC cells (MKN45 and MKN74) transfected with *OSBPL3*-specific siRNAs, according to RT-qPCR (left panel) and western blotting (right panel), respectively. si: siRNA targeting *OSBPL3*. $N = 3$, error bars represent the mean ± SD.

c. *OSBPL3* mRNA and protein expression in GC cells (MKN45) transfected with shRNAs, according to RT-qPCR (left panel) and western blotting (right panel), respectively. sh: shRNA targeting *OSBPL3*. $N = 3$, error bars represent the mean ± SD.

Supplementary Figure S3.

MTT assays in MKN45 cells transfected with *OSBPL3*-specific shRNA. sh: shRNA targeting *OSBPL3*. $N = 9$, error bars represent the mean ± SD. Student's *t*-test.

Figure S1

a.

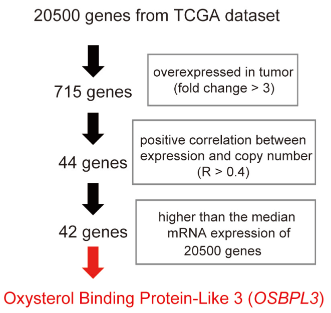

c.

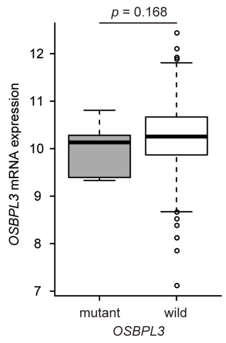

b.

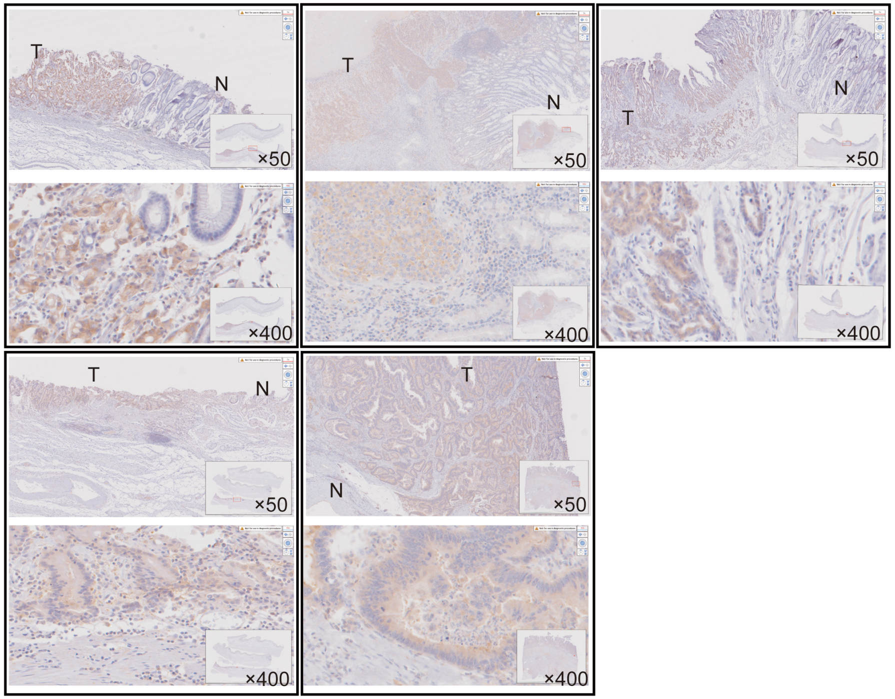

Figure S2

a.

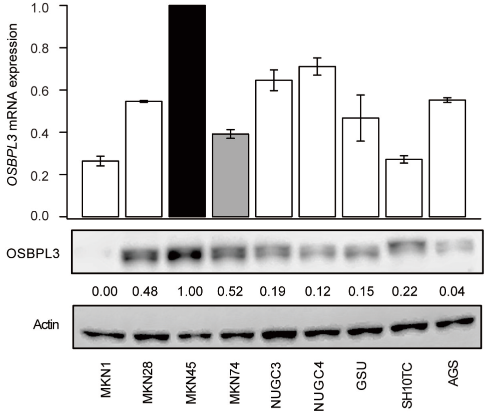

b.

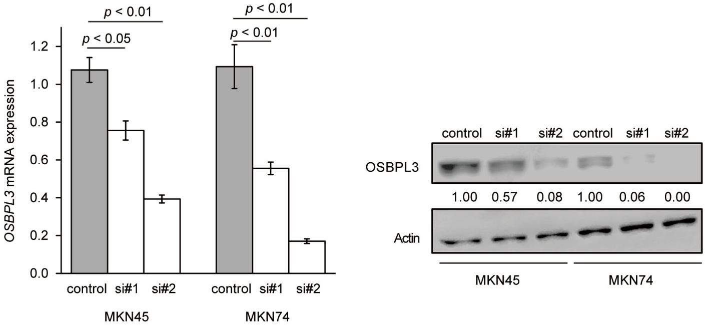

c.

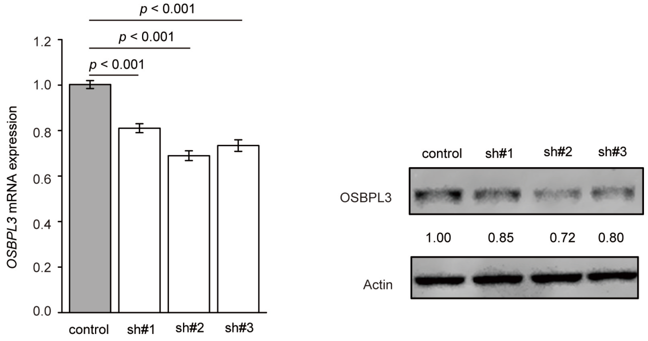

Figure S3

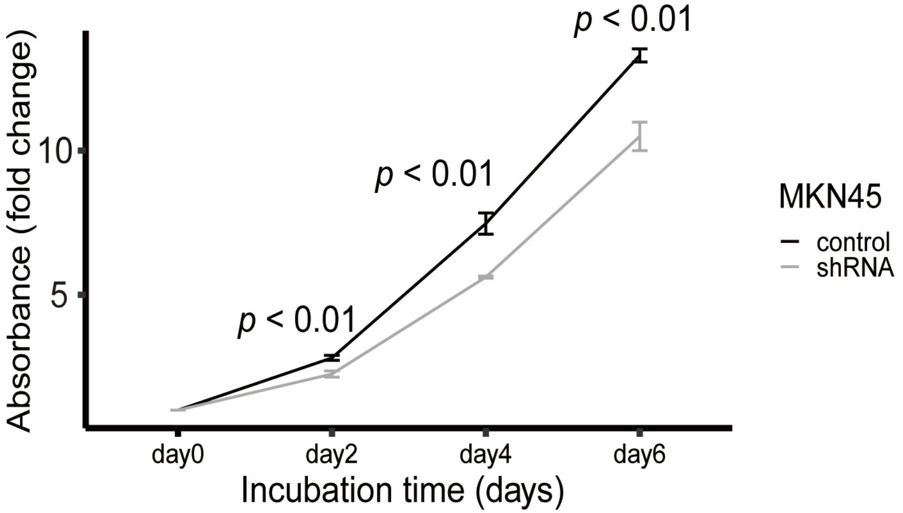

p < 0.01
p < 0.01
p < 0.01
Absorbance (fold change)
10
5
day0
day2
day4
day6
Incubation time (days)
MKN45
control
shRNA

SUPPLEMENTARY TABLE LEGENDS

Supplementary Table S1. The list of downregulated genes in MKN45 cells transfected with *OSBPL3*-specific short hairpin RNA (*OSBPL3*-shRNA) relative to control cells (fold change < 0.5).

Supplementary Table S2. The sequences of the siRNAs and shRNAs targeting *OSBPL3* used in this study.

Supplementary Table S3. *OSBPL3* mRNA expression and clinicopathological factors of GC cases from the GSE15459 dataset (n = 192).

Table S1

downgenes																				
A4GALT	AQP11	C15orf65	CCDC77	CYSTM1	FAM101A	GBX2	HOXA9	KDM7A	LNP1	LRRC46	MRGPRF	OASL	PPAN-P2RY11	RIBC1	SLC25A42	SNORA68	SYT7	TPM2	ULK2	ZNF30
AADAC	ARHGEF10L	C19orf18	CCDC96	DACT2	FAM109A	GCNT2	HOXB8	KDM8	LNX1	LRRC49	MRPS31P5	OAZ3	PPAP2B	RILP	SLC27A3	SNORA74A	SYTL2	TRABD2A	UNC50	ZNF438
AAK1	ARL14EP	C19orf54	CCRL2	DEFB123	FAM122C	GCSHP3	HOXC-AS1	KIAA1147	LOC100134868	LTB4R2	MSMB	OR7E91P	PPARA	RIMS3	SLC27A5	SNORA76C	TBX15	TRAM2-AS1	UNC5CL	ZNF460
ABCC6P2	ARL9	C19orf81	CDH17	DEPDC5	FAM156B	GDPD3	HOXC12	KIAA1211	LOC100288162	LUM	MSMP	ORAOV1	PQLC2	RNF125	SLC28A3	SNORA8	TCEA3	TRAPPC2P1	USPL1	ZNF510
ABHD8	ARMC3	C19orf82	CDKN2C	DGUOK-AS1	FAM185A	GEMIN6	HSD17B2	KIAA1841	LOC100288748	MAP3K7CL	MSX1	OSBPL3	PRICKLE4	RNF166	SLC31A2	SNORA9	TCHP	TRERF1	VAMP5	ZNF565
ABLIM3	ASB9	C1QL1	CEBPA	DIAPH2	FAM222A	GLYCTK	IFI27	KIAA1919	LOC100289361	MAPK11	MT1DP	OSBPL5	PRKCQ-AS1	RNF207	SLC35D1	SNORD10	TEF	TRIM15	VIM	ZNF586
ACADSB	ASGR1	C22orf23	CETN4P	DISP2	FAM45B	GNPTAB	IFI27L1	KIF27	LOC100506314	MATN1-AS1	MT1F	OTUD1	PRTFDC1	RNF32	SLC39A14	SOAT1	TEKT2	TRIM24	VIPR1	ZNF611
ACTRT3	ATF2	C2orf70	CHCHD4	DMBX1	FAM72A	GPR75-ASB3	IFI27L2	KITLG	LOC100507002	MATN2	MYO1A	OXNAD1	PSAPL1	ROR1	SLC39A5	SP4	TENC1	TRIM31	VMAC	ZNF619
ADCY9	ATP2B1	C2orf82	CIDEC	DNAJB14	FAM81A	GSTA1	IFITM10	KLC3	LOC100996324	MBLAC1	MYO7B	P4HA2-AS1	PSMB9	RPL21	SLC45A4	SPAG4	TERT	TRIM47	VSIG1	ZNF684
ADH6	AXIN1	C5orf66	CIDECP	DND1	FAM83B	GSTA4	IGFBP6	KLF2	LOC100996437	MBNL3	MYPOP	PABPC3	PSMG4	RPS6KA5	SLC7A5P2	SPATA7	TFAP2E	TRIM52	WDPCP	ZNF761
ADM5	B3GNT9	C6orf147	CLDN22	DPH3P1	FAM83E	HAGH	IGFBP7	KLF4	LOC101926888	MCF2L-AS1	NAGS	PARP11	PTGS2	RRNAD1	SLC9A2	SPEF2	TFEB	TRIQK	WDR7	ZNF785
ADPRHL1	B3GNTL1	C6orf203	CLRN3	DPH6	FAM86B2	HARBI1	IGFLR1	KLHL13	LOC101927572	MCM3AP-AS1	NANOS3	PAXIP1OS	PTPLA	RTN2	SLC9A3R2	SPINK1	TFF1	TRMT61B	WNT10B	ZP3
AGER	B4GALNT1	C7orf13	CLU	DPY19L2P4	FAM89A	HCN3	IL15RA	KRTCAP3	LOC101927755	MED14OS	NAPA-AS1	PCAT6	PTPRE	RTN4RL1	SLC9B2	SPINK4	THAP8	TSACC	WNT7A	ZRANB3
AGO3	BAAT	C7orf57	CNFN	DUSP10	FASTKD1	HDHD1	IL18	L3HYPDH	LOC101928062	MED4-AS1	NAT6	PCSK5	PTPRH	RWDD2A	SLCO2B1	SPON1	THNSL1	TSC22D3	WWOX	ZSCAN16-AS1
AHCYL2	BCL2	C9orf163	CNTRL	DUSP18	FCRLB	HDHD3	IL1R2	LACE1	LOC101928784	MEF2B	NDST2	PDGFA	PXDC1	S100A14	SLFN11	SPRR1B	TIGD1	TSPYL5	XYLT1	
AK8	BCL2L11	C9orf64	COLCA1	DUSP28	FGFR2	HECTD2	IL1RN	LBX2	LOC101929767	MEGF9	NDUFC2-KCTD	PGBD4	QRFP	SAMD12	SLMO1	SPSB3	TLDC2	TTC28-AS1	ZBED5-AS1	
AKR1B10	BCYRN1	C9orf72	CORO2A	DYRK1B	FGFR3	HELB	IL22RA1	LCORL	LOC102723354	MFI2-AS1	NEK9	PHF7	RAB27B	SAPCD1	SMARCA5-AS1	SRGAP2B	TLN2	TTC32	ZBTB46	
AKR7L	BIK	CA2	CREB3L3	DYX1C1-CCPG	FGGY	HEPH	IL2RG	LEPREL1	LOC102723373	MFSD4	NFE2	PHGR1	RAB4B	SCARNA1	SMG1P5	SRGAP2C	TM4SF1-AS1	TUBAL3	ZBTB5	
ALDH1A1	BIRC2	CABP1	CRELD1	EEFSEC	FLVCR1-AS1	HERC6	IL4R	LGALS8	LOC102800310	MGAT3	NIPAL1	PIGB	RAB8B	SCARNA22	SMIM14	SSSCA1-AS1	TM4SF4	TUBD1	ZC2HC1C	
AMICA1	BIVM-ERCC5	CACNA2D4	CROCCP2	EFNA2	FMO5	HIST1H4K	IQCJ-SCHIP1	LGR5	LOC113230	MIA	NIPAL4	PIGK	RAET1E-AS1	SEC14L2	SMIM19	STAG3L1	TMCC3	TUBG2	ZCWPW1	
AMN	BLNK	CALML4	CSAD	EIF3CL	FNDC8	HIST2H2AA4	IQGAP2	LHX9	LOC158960	MIOS	NKD1	PIH1D2	RANBP6	SELM	SNAPC2	STBD1	TMEM107	TUSC1	ZDHHC8P1	
AMN1	BLOC1S5-TXNI	CAMK2N1	CSF2RA	ELAC1	FOXD2	HIST2H2BC	IRF8	LIN52	LOC220729	MIR1307	NME5	PIK3AP1	RASA2	SEMA3B	SNHG9	STRN	TMEM110-MUS	TWIST1	ZFPM1	
ANKHD1-EIF4E	BMP8B	CAP2	CSRP2BP	EPB41L1	FRAT1	HIST2H2BF	IRX2	LINC00460	LOC400655	MIR22HG	NMNAT3	PITPNA-AS1	RASL10B	SETD6	SNORA16B	STS	TMEM171	TYMSOS	ZFYVE28	
ANKRD23	BMS1P17	CARD8	CTIF	EPHB2	FRG1B	HLA-G	ITPKA	LINC00491	LOC400927	MIR941-1	NMRK1	PLEKHG2	RASL11A	SGK1	SNORA27	STX2	TMEM187	UBE2Q2P2	ZNF17	
ANTXR2	BSCL2	CASK	CTSE	EPS8L1	FUZ	HMMR-AS1	JHDM1D-AS1	LINC00526	LOC439994	MIS18A	NPNT	PLEKHO1	RBM12B-AS1	SH3RF3-AS1	SNORA32	SULT2B1	TMEM53	UBE2W	ZNF185	
AOC1	BST2	CCDC102A	CTSF	ERICH5	FZD2	HMOX1	JOSD2	LINC00675	LOC728323	MMP28	NR1I2	PLK4	RCBTB1	SH3YL1	SNORA40	SUMO1P3	TMEM92	UBE3B	ZNF2	
AP4S1	C10orf11	CCDC107	CWF19L2	ETS1	GAB2	HOXA10-AS	KALRN	LINC01003	LOC730101	MOB3B	NR4A1	PMF1-BGLAP	REG4	SIX3-AS1	SNORA51	SUV420H2	TMSB15B	UCHL1	ZNF20	
APITD1-CORT	C11orf35	CCDC149	CXCL17	ETV7	GALNT18	HOXA13	KBTBD11	LINC01018	LONRF3	MOGAT2	NUDT16P1	PMM1	REP15	SLC16A1	SNORA59B	SWSAP1	TNFRSF6B	UFSP1	ZNF204P	
APOBEC1	C11orf53	CCDC159	CYP2S1	EVA1B	GAN	HOXA5	KCNF1	LINC01311	LRP1	MOSPD2	NUDT7	PMS2L2	REPS2	SLC16A1-AS1	SNORA6	SYBU	TNIK	UGT1A1	ZNF211	
ZSCAN26	C12orf73	CCDC74B	CYP4F12	EVI5	GATA6	HOXA6	KDELC1	LMO4	LRRC37B	MPP1	NUMBL	PMS2P4	RGS14	SLC17A9	SNORA67	SYDE2	TNNI1	ULBP2	ZNF252P	

Table S2

	Nucleotide Sequence(5' to 3')	Nucleotide Sequence(5' to 3')
siOSBPL3#1	Sense:CCUUGAUAGUGGUCGGGAAtt	Antisense:UUCCCGACCACUAUCAAGGac
siOSBPL3#2	Sense:GCUUUCUAAUGAAAGUAGAtt	Antisense:UCUACUUUCAUUAGAAAGCtg
	Nucleotide Sequence(5' to 3')	
shOSBPL3#1-top	TGCTGATCACTGAGAGCCCGACATCAGTTTTGGCCACTGACTGACTGATGTCGCTCTCAGTGAT	
shOSBPL3#1-bottom	CCTGATCACTGAGAGCGACATCAGTCAGTCAGTGGCCAAAACTGATGTCGGGCTCTCAGTGATC	
shOSBPL3#2-top	TGCTGCCAAGAACCGGATTAAATGGCGTTTTGGCCACTGACTGACGCCATTTACCGGTTCTTGG	
shOSBPL3#2-bottom	CCTGCCAAGAACCGGTAAATGGCGTCAGTCAGTGGCCAAAACGCCATTTAATCCGGTTCTTGGC	
shOSBPL3#3-top	TGCTGCTAAGTTCCAAATAGGTGCCGGTTTTGGCCACTGACTGACCGGCACCTTTGGAACTTAG	
shOSBPL3#3-bottom	CCTGCTAAGTTCCAAAGGTGCCGGTCAGTCAGTGGCCAAAACCGGCACCTATTTGGAACTTAGC	

Table S3 *OSBPL3* mRNA expression and clinicopathological factors of GC cases from the GSE15459 dataset (n = 192).

Factors	High expression (n = 44)	Low expression (n = 148)	P value
Age (mean ± SD)	67.6 ± 12.7	63.4 ± 13.3	0.06
Female sex	11 (26%)	56 (38%)	0.15
Histological type (well- or moderately differentiated adenocarcinoma)	22 (50%)	98 (67%)	< 0.05
Depth of tumor invasion (≥ SS)	31 (84%)	87 (64%)	< 0.05
Lymph node metastasis (+)	34 (92%)	102 (75%)	< 0.05
Distant metastasis (+)	7 (19%)	21 (15%)	0.62
pStage (≥ III)	36 (82%)	96 (65%)	< 0.05
Pylori infection (+)	13 (65%)	44 (64%)	1.00

SD, standard deviation; SS, subserosa; Pylori, helicobacter pylori; pStage, the 6th UICC staging system;

Analysis of TCGA

We obtained mRNA expression, DNA copy number alteration, and somatic mutation data from 443 GC patients from the Firehose pipeline at the Broad Institute. We also used Firehose to obtain mRNA expression and clinical assessment data from 408 bladder cancer (bladder), 1093 breast cancer (breast), 304 cervical cancer (cervical), 36 cholangiocarcinoma (bile_duct), 454 colon cancer (colon), 184 esophageal cancer (esophagus), 520 head and neck cancer (head_neck), 533 kidney clear cell carcinoma (kidney_clear), 290 kidney papillary cell carcinoma (kidney_papillary), 371 liver cancer (liver), 515 lung adenocarcinoma (lung_ad), 501 lung squamous cell carcinoma (lung_sc), 304 ovarian cancer (ovarian), 178 pancreatic cancer (pancreas), 497 prostate cancer (prostate), 166 rectal cancer (rectum), 501 thyroid carcinoma (thyroid), and 545 uterine cancer (uterin) patients. The mRNA expression data (FPKM values, raw counts) were subjected to quantile normalization. A DNA copy number (log 2(N/2)) < 0 was defined as a loss, equal to 0 as no change (neutral), and > 0 as a gain.

The link of the Broad Institute's Firehose

(http://gdac.broadinstitute.org/runs/stddata__2016_01_28/data/STAD/20160128/).

(http://gdac.broadinstitute.org/runs/stddata__2016_01_28/data/BLCA/20160128/),

(http://gdac.broadinstitute.org/runs/stddata__2016_01_28/data/BRCA/20160128/),

(http://gdac.broadinstitute.org/runs/stddata__2016_01_28/data/CESC/20160128/),

(http://gdac.broadinstitute.org/runs/stddata__2016_01_28/data/CHOL/20160128/),

(http://gdac.broadinstitute.org/runs/stddata__2016_01_28/data/COAD/20160128/),

(http://gdac.broadinstitute.org/runs/stddata__2016_01_28/data/ESCA/20160128/),

(http://gdac.broadinstitute.org/runs/stddata__2016_01_28/data/HNSC/20160128/),

(http://gdac.broadinstitute.org/runs/stddata__2016_01_28/data/KIRC/20160128/),

(http://gdac.broadinstitute.org/runs/stddata__2016_01_28/data/KIRP/20160128/),

(http://gdac.broadinstitute.org/runs/stddata__2016_01_28/data/LIHC/20160128/),

(http://gdac.broadinstitute.org/runs/stddata__2016_01_28/data/LUAD/20160128/),

(http://gdac.broadinstitute.org/runs/stddata__2016_01_28/data/LUSC/20160128/),

(http://gdac.broadinstitute.org/runs/stddata__2016_01_28/data/OV/20160128/),
(http://gdac.broadinstitute.org/runs/stddata__2016_01_28/data/PAAD/20160128/),
(http://gdac.broadinstitute.org/runs/stddata__2016_01_28/data/PRAD/20160128/),
(http://gdac.broadinstitute.org/runs/stddata__2016_01_28/data/READ/20160128/),
(http://gdac.broadinstitute.org/runs/stddata__2016_01_28/data/THCA/20160128/),
(http://gdac.broadinstitute.org/runs/stddata__2016_01_28/data/UCEC/20160128/).

GSE15459 dataset

This dataset contained no mRNA expression data from normal tissues. The mRNA expression data from 200 tumor tissues were subjected to quantile normalization. Clinical data were available from 192 of the patients. We used this GC dataset to evaluate the prognostic significance of *OSBPL3* expression in GC patients. The GC patients were divided according to their *OSBPL3* expression level into high and low expression groups using the minimum *p*-value approach. This approach is a comprehensive method to identify the optimal risk separation cut-off point in continuous gene expression measurements for survival analyses in multiple datasets.

Cancer Cell Line Encyclopedia (CCLE) dataset

We downloaded mRNA expression data from 1037 cell lines and DNA copy number alteration data from 1042 cell lines in the CCLE (https://portals.broadinstitute.org/ccle/home). A DNA copy number (log 2(N/2)) < 0 was defined as a loss, equal to 0 as no change (neutral), and > 0 as a gain.

Figure S2a

original WB images

Marker

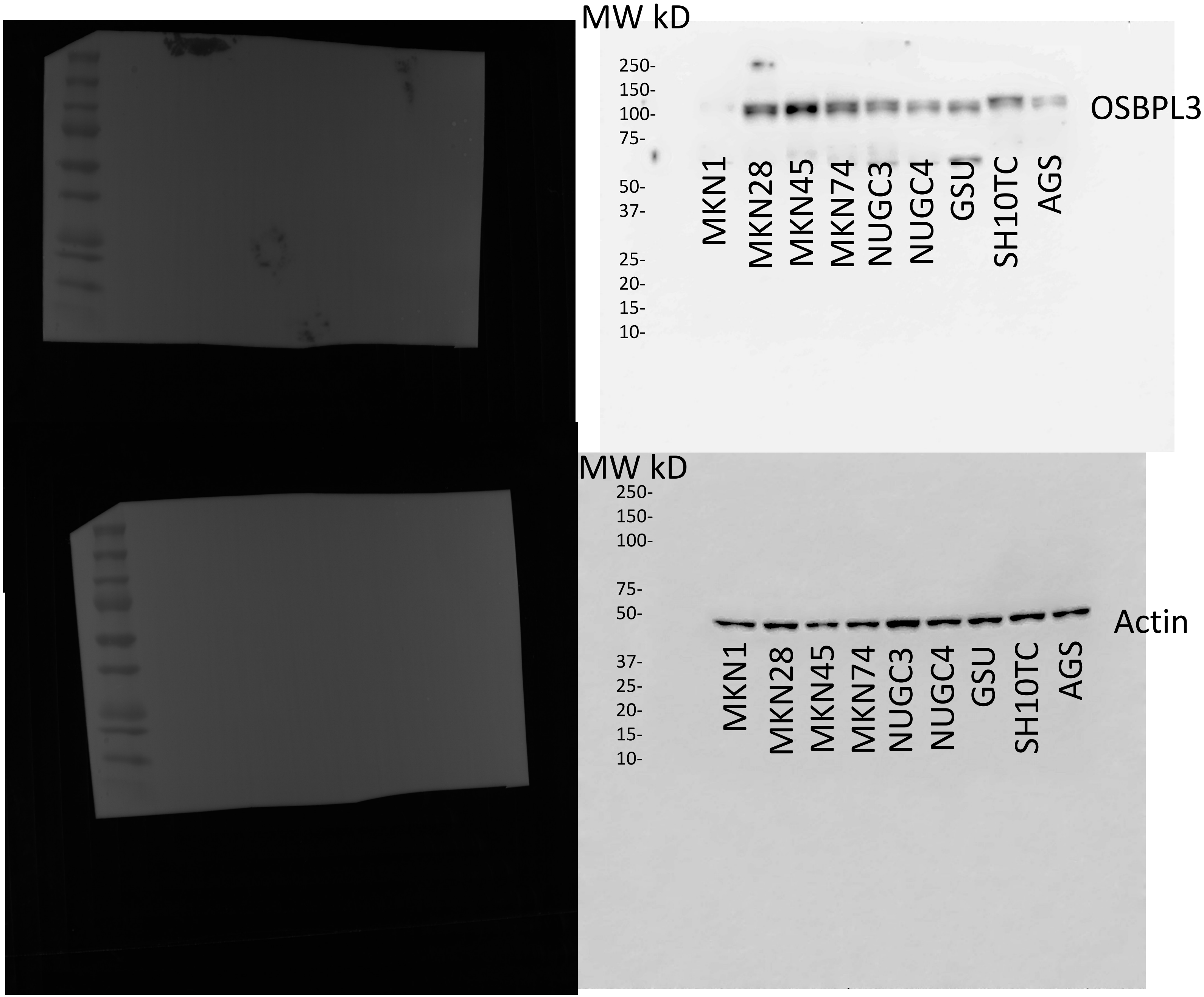

Figure S2b

original WB images

Marker

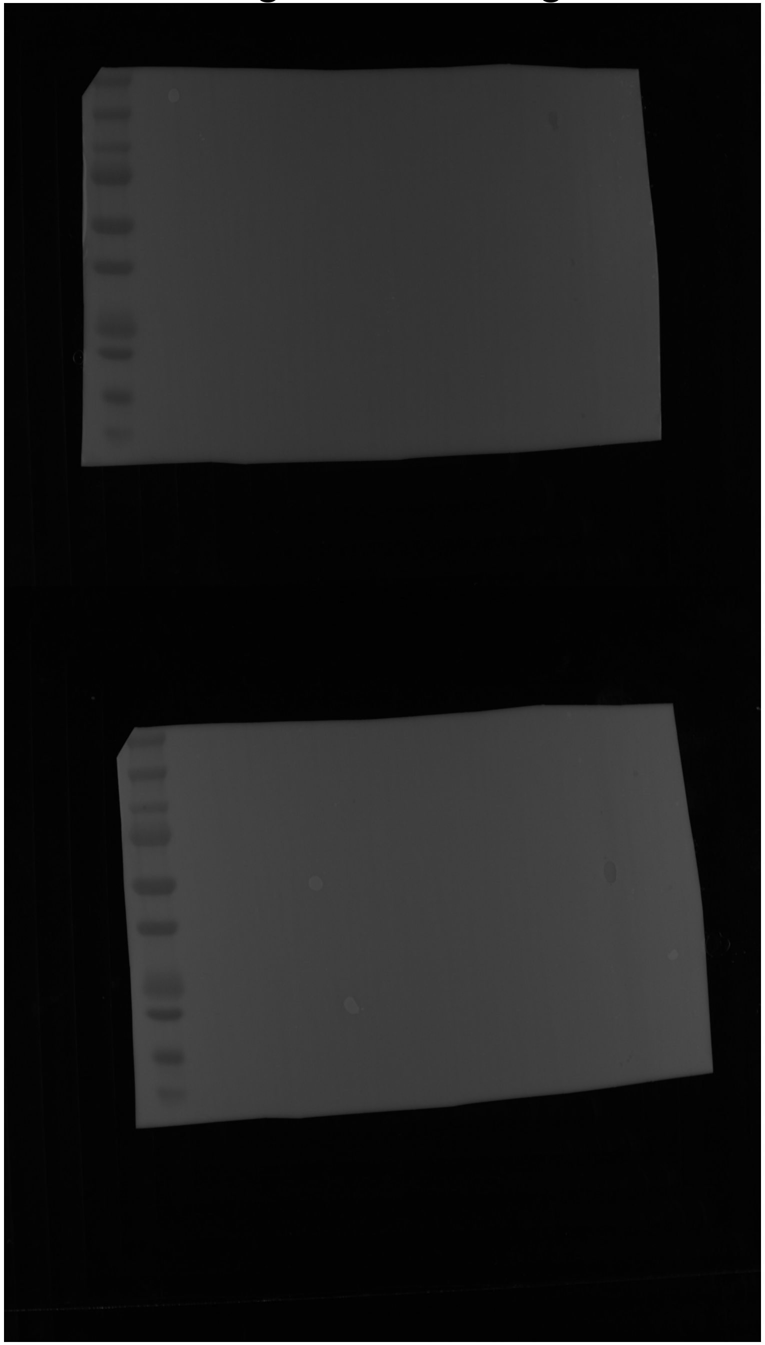

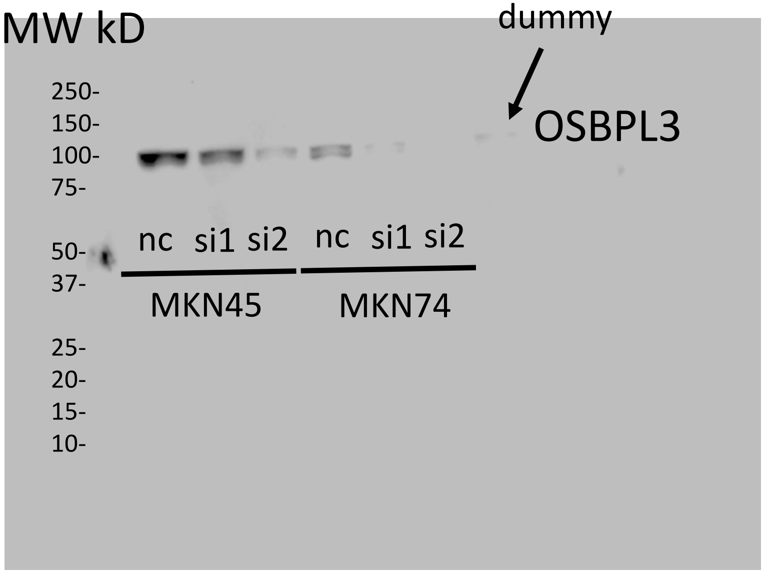

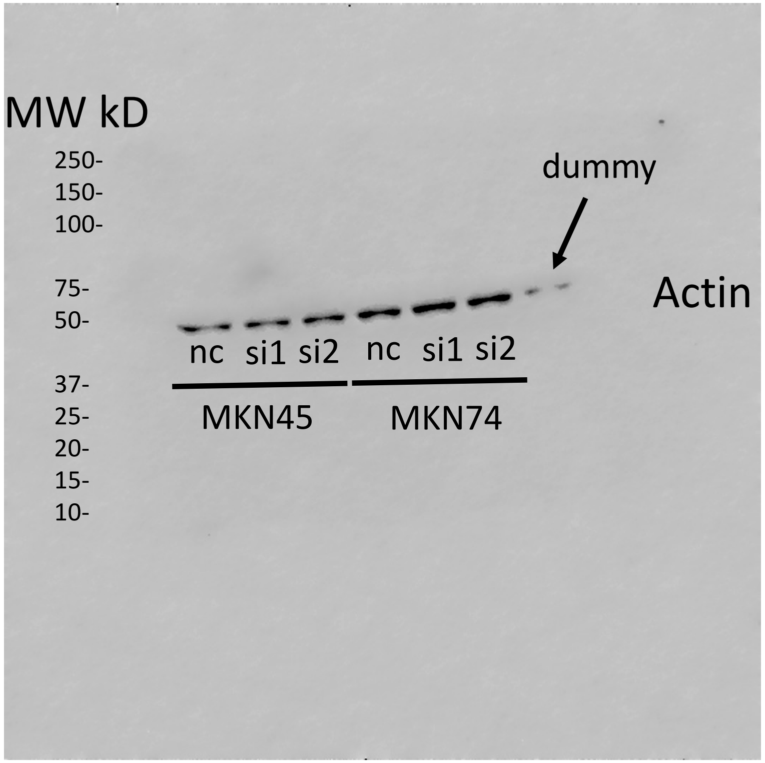

Figure S2c

original WB images

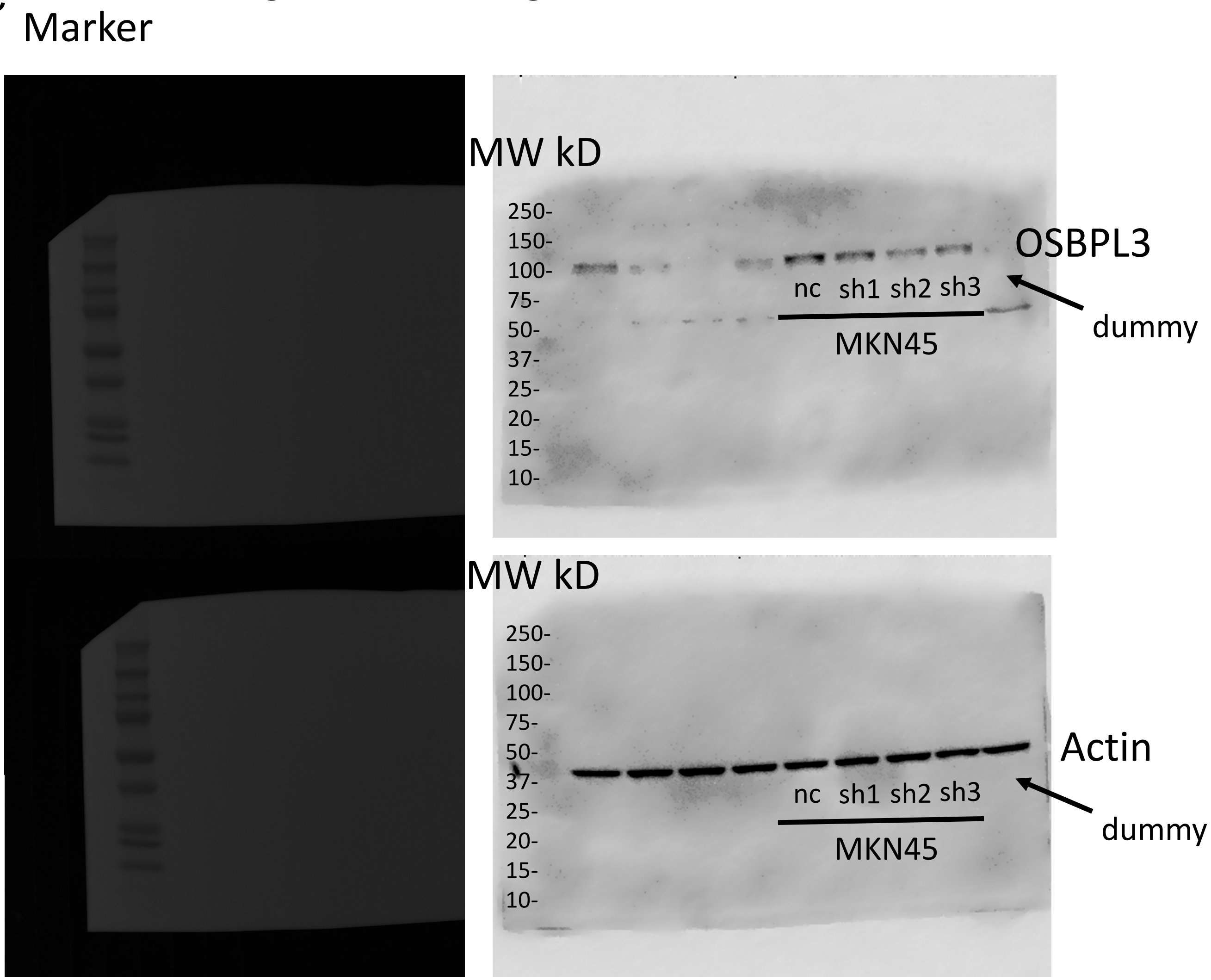

Figure 3c---MKN45 original WB images

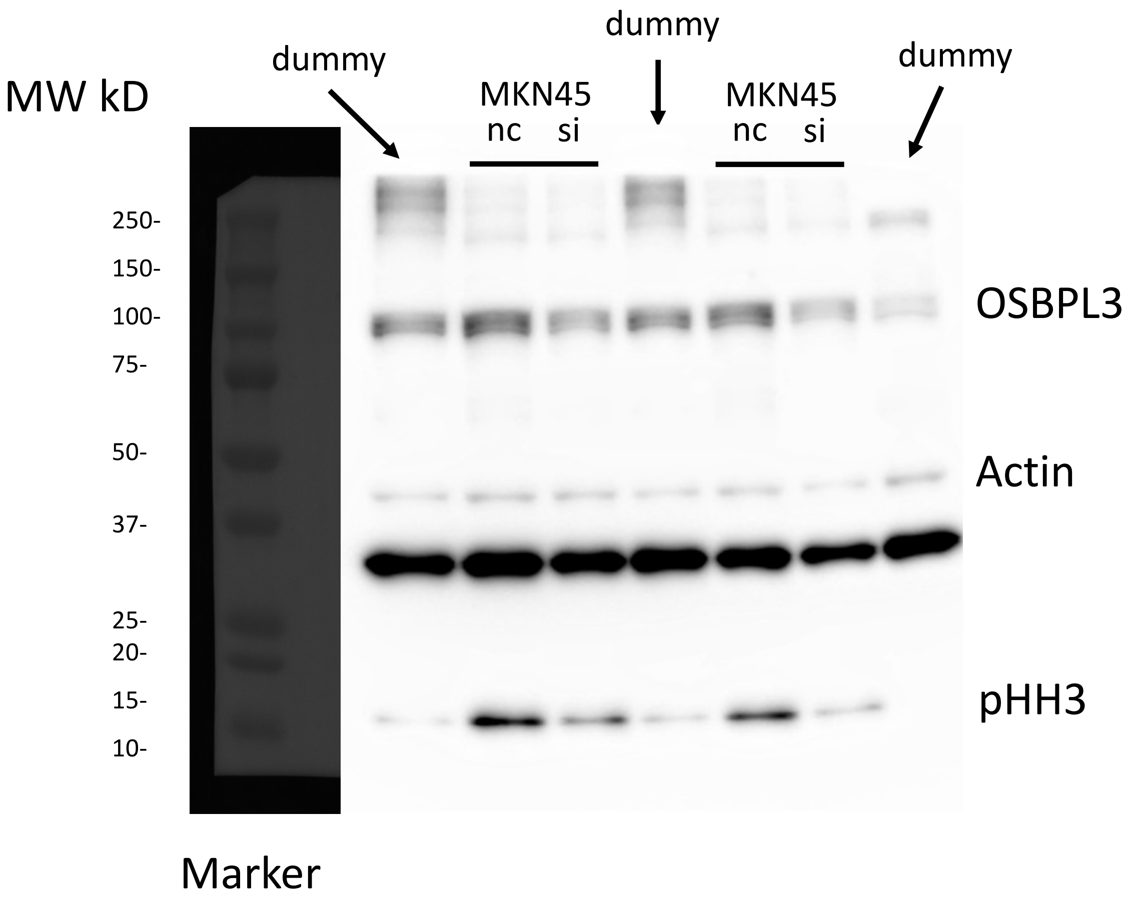

Figure 3c---MKN74

original WB images

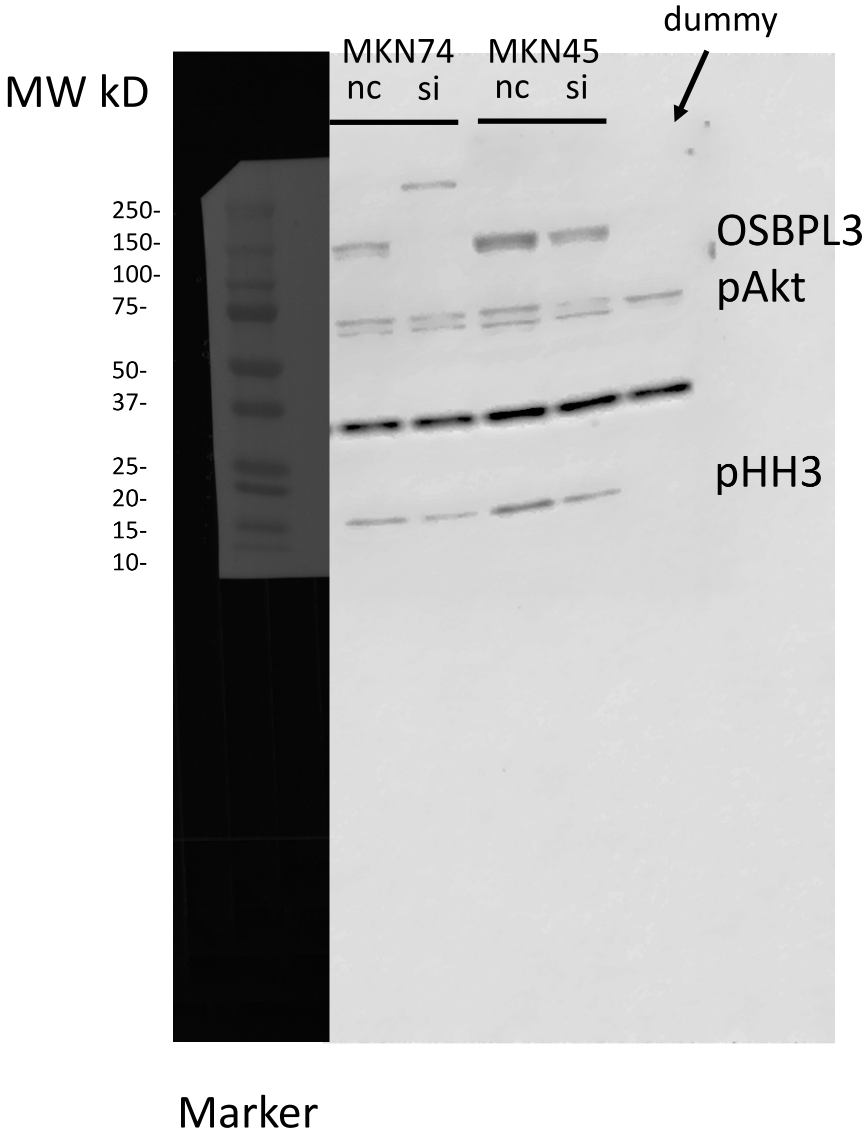

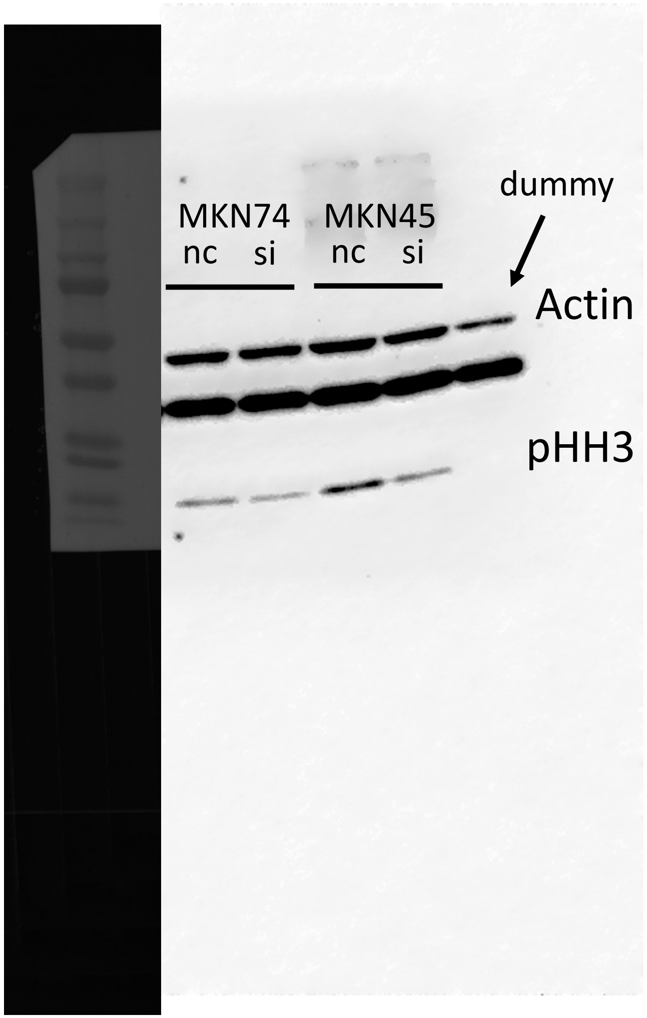

original WB images

Figure 4b

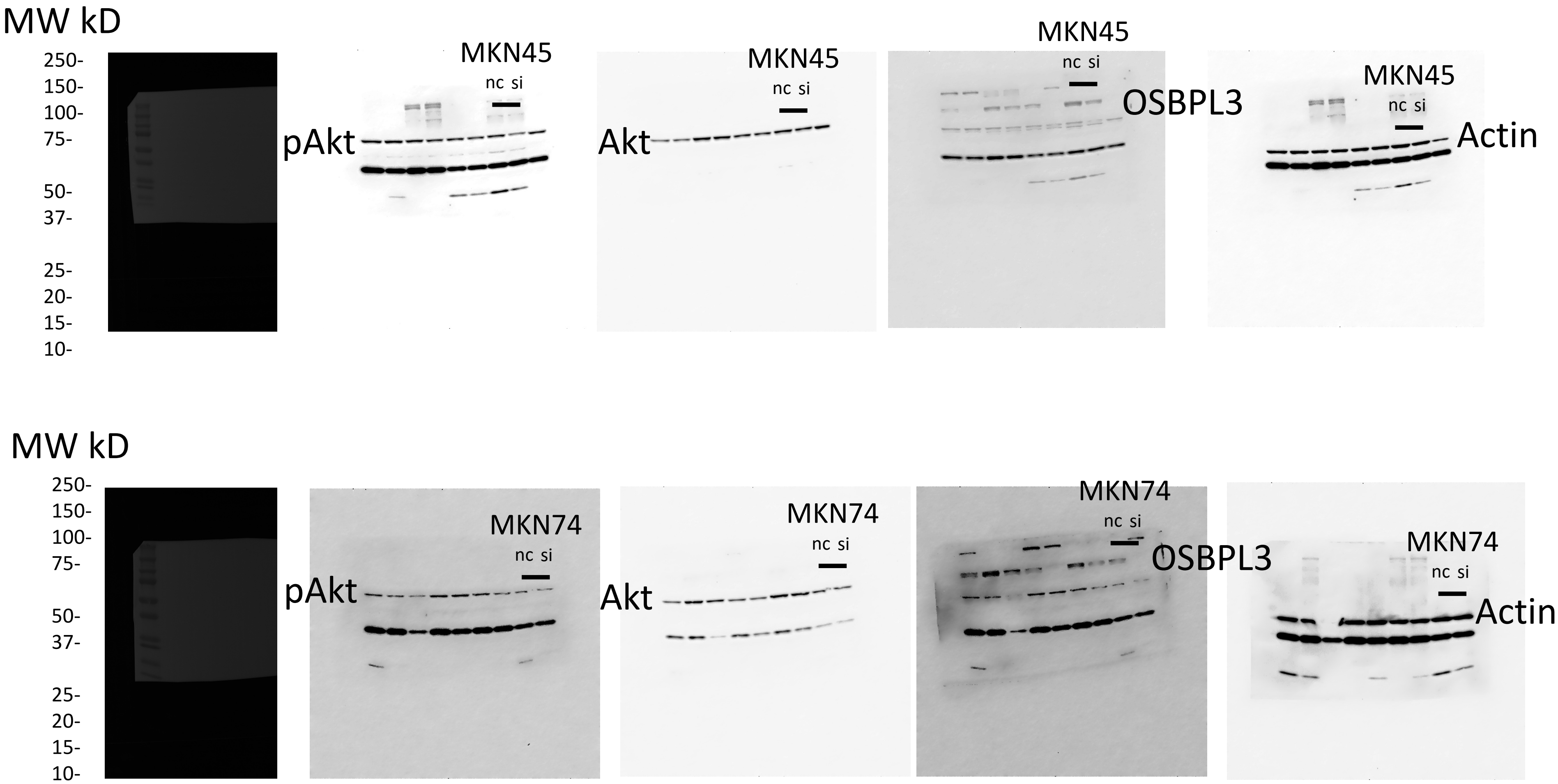

Figure 4c---MKN45 original WB images

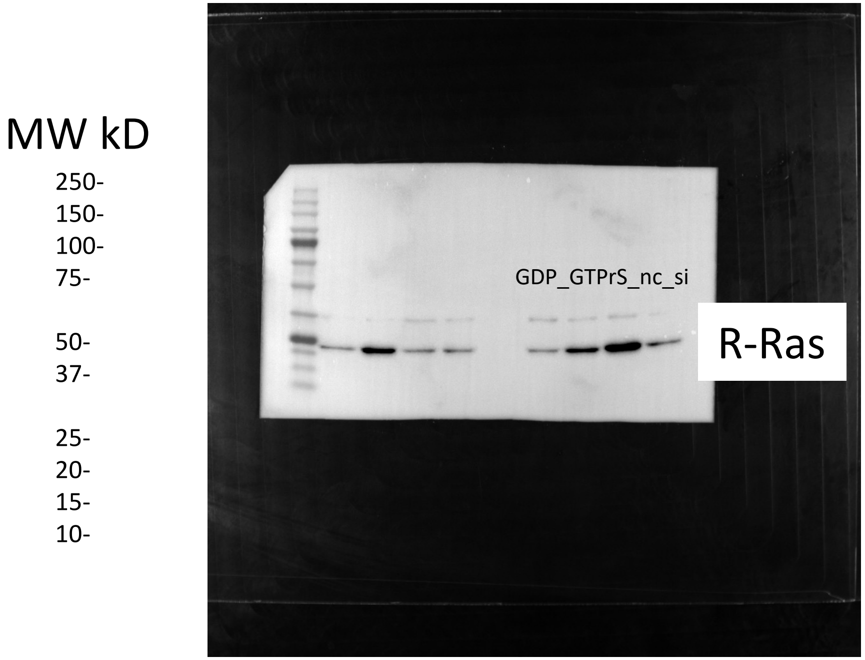

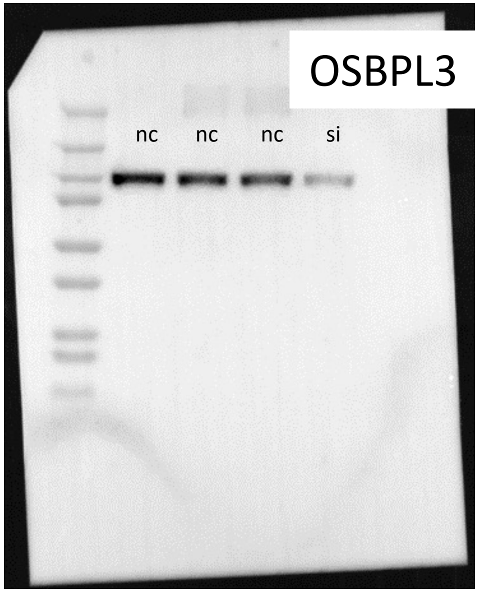

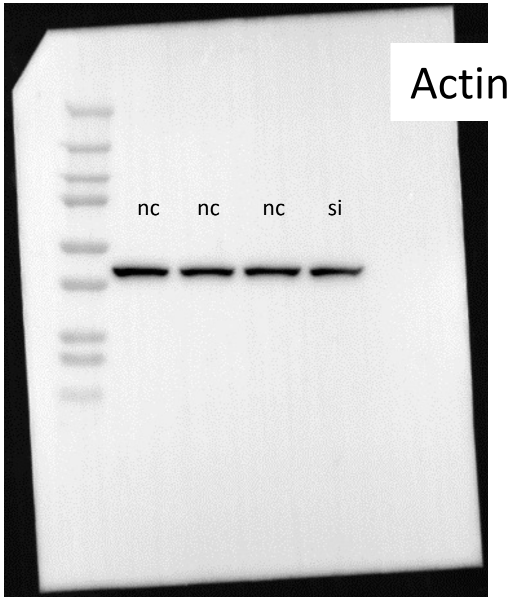

Figure 4c---MKN74 original WB images

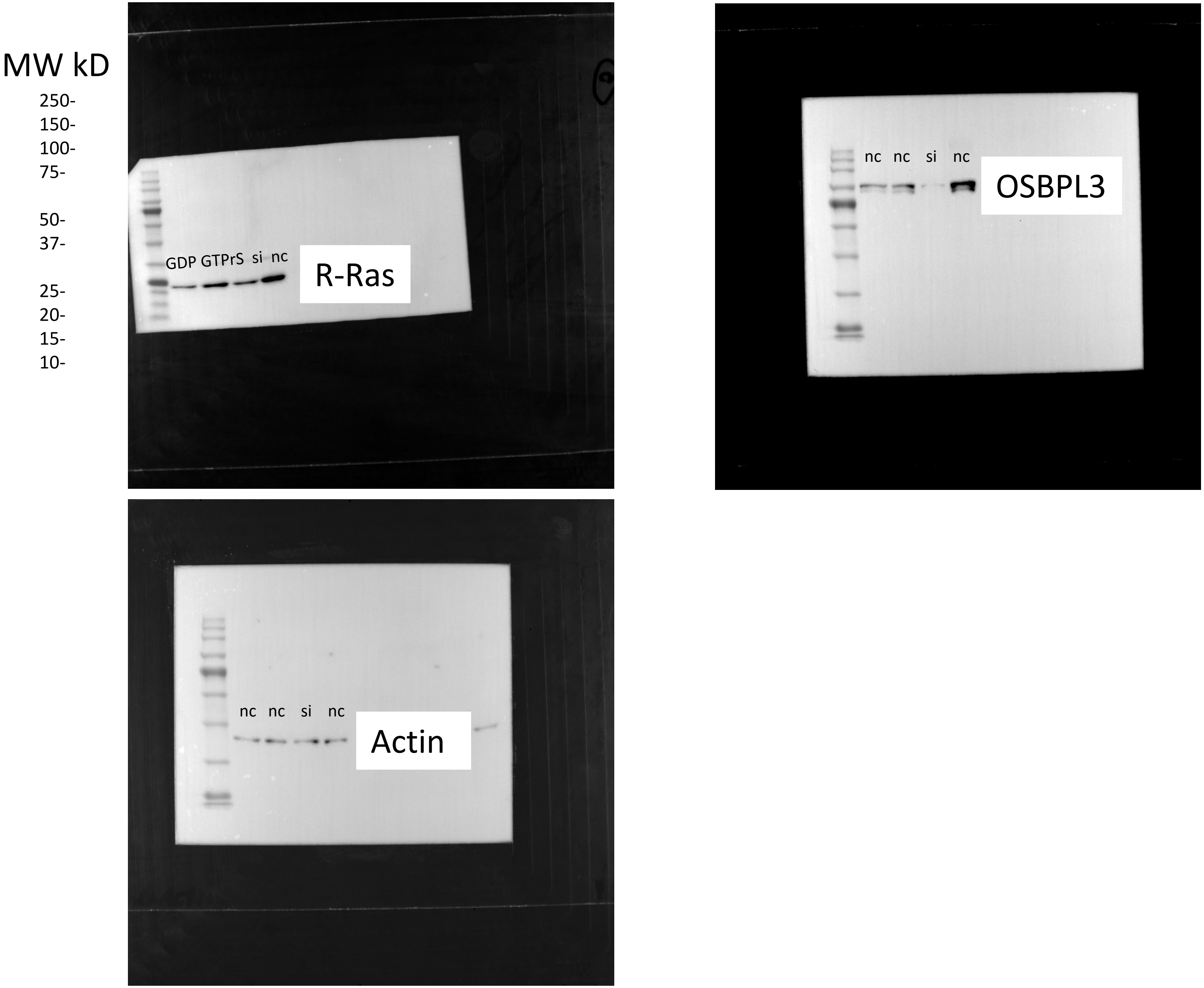

Supplement: Supplementary file 1 — Supplementary Information. [file 41598_2021_98485_MOESM1_ESM.pdf]
